# BSGatlas: a unified *Bacillus subtilis* genome and transcriptome annotation atlas with enhanced information access

**DOI:** 10.1099/mgen.0.000524

**Published:** 2021-02-04

**Authors:** Adrian Sven Geissler, Christian Anthon, Ferhat Alkan, Enrique González-Tortuero, Line Dahl Poulsen, Thomas Beuchert Kallehauge, Anne Breüner, Stefan Ernst Seemann, Jeppe Vinther, Jan Gorodkin

**Affiliations:** ^1^​ Center for Non-coding RNA in Technology and Health, Department of Veterinary and Animal Sciences, University of Copenhagen, 1871 Frederiksberg, Denmark; ^2^​ Division of Oncogenomics, Netherlands Cancer Institute, 1066 CX Amsterdam, The Netherlands; ^3^​ Section for Computational and RNA Biology, Department of Biology, University of Copenhagen, 1165 Copenhagen, Denmark; ^4^​ Novozymes, Bagsværd, Denmark; ^†^​ Present address: School of Science, Engineering and Environment, University of Salford, Salford, UK

**Keywords:** *B. subtilis*, genome annotation, non-coding and structured RNAs, operons

## Abstract

A large part of our current understanding of gene regulation in Gram-positive bacteria is based on *Bacillus subtilis *, as it is one of the most well studied bacterial model systems. The rapid growth in data concerning its molecular and genomic biology is distributed across multiple annotation resources. Consequently, the interpretation of data from further *B. subtilis* experiments becomes increasingly challenging in both low- and large-scale analyses. Additionally, *B. subtilis* annotation of structured RNA and non-coding RNA (ncRNA), as well as the operon structure, is still lagging behind the annotation of the coding sequences. To address these challenges, we created the *B. subtilis* genome atlas, BSGatlas, which integrates and unifies multiple existing annotation resources. Compared to any of the individual resources, the BSGatlas contains twice as many ncRNAs, while improving the positional annotation for 70 % of the ncRNAs. Furthermore, we combined known transcription start and termination sites with lists of known co-transcribed gene sets to create a comprehensive transcript map. The combination with transcription start/termination site annotations resulted in 717 new sets of co-transcribed genes and 5335 untranslated regions (UTRs). In comparison to existing resources, the number of 5′ and 3′ UTRs increased nearly fivefold, and the number of internal UTRs doubled. The transcript map is organized in 2266 operons, which provides transcriptional annotation for 92 % of all genes in the genome compared to the at most 82 % by previous resources. We predicted an off-target-aware genome-wide library of CRISPR–Cas9 guide RNAs, which we also linked to polycistronic operons. We provide the BSGatlas in multiple forms: as a website (https://rth.dk/resources/bsgatlas/), an annotation hub for display in the UCSC genome browser, supplementary tables and standardized GFF3 format, which can be used in large scale -omics studies. By complementing existing resources, the BSGatlas supports analyses of the *B. subtilis* genome and its molecular biology with respect to not only non-coding genes but also genome-wide transcriptional relationships of all genes.

## Data Summary

The provided genome annotation BSGatlas is relative to the RefSeq reference assembly of *Bacillus subtilis* 168 (accession no. ASM904v1). All utilized external data, the resulting annotation and the BSGatlas generating scripts are permanently stored at https://doi.org/10.5281/zenodo.4305872. The BSGatlas is available for download in the standardized GFF3 format (https://doi.org/10.5281/zenodo.4305869) or as a UCSC genome browser hub at https://rth.dk/resources/bsgatlas/. The genomic coordinates of all BSGatlas annotations in the form of supplementary tables are also available with the online version of this article.

**Impact Statement**

Current annotation efforts of the *Bacillus subtilis* genome are predominately focused on coding sequences; however, non-coding genes and structured RNA elements fulfil essential functions in the organism. However, access to the non-coding annotation is challenging. In order to find such elements and their regulatory partners, transcript annotations that include the untranslated regions (UTRs) are needed. Unfortunately, information about transcription start/termination sites (which give rise to UTRs) is found in different databases than those that contain information about structured RNA. Here, we integrate multiple databases and annotation resources of different annotation types to address the multiple databases access problem. We provide the resulting annotation in multiple formats to increase its utility. This large-scale integration provided new UTR annotations, which resulted in a substantial increase in number. We further inspected isoform transcripts and investigated the complex operon architecture of *B. subtilis *. To make the integration process transparent, we show the information from the individual databases in a genome browser.

## Introduction

*Bacillus subtilis* (* Firmicutes *, *Bacilli *) is a Gram-positive soil micro-organism that is central for multiple research fields. It is widely used as a model system for the study of gene regulation and it is probably the best-studied bacterial species apart from *Escherichia coli *. In industrial applications, it is used as a host organism for the production of enzymes and other proteins [1]. *B. subtilis* is actively studied with modern high-throughput -omics methods, such as RNA-seq [2, 3], which already provided important novel insights into gene regulation and demonstrated that bacterial transcription is even more complex than previously expected [4, 5]. However, high-throughput analyses of gene expression data and interpretation with respect to biological function are highly dependent on the quality of genome annotations. The *B. subtilis* genome annotation is, as for many other genomes, subject of ongoing, manual expert curation [6], which requires substantial and non-trivial efforts [6, 7]. However, the current annotations of *B. subtilis* focus on protein coding sequences (CDSs) [8], insofar that although many genome coordinate annotations exist for structured RNA elements, non-coding RNA (ncRNA) genes and untranslated regions (UTRs) of mRNAs, these annotations are challenging to access; in particular for high-throughput access. Consequently, full mRNA transcripts are rarely annotated, which constrains the study of post-transcriptional regulation.

Bacterial genomes contain operons, such that multiple genes are transcribed from one promoter region in a single RNA molecule [9, 10]. Besides the 5′ and 3′ UTRs, the mRNAs also have internal UTRs [9]. The lengths of transcribed UTRs can differ, depending on the isoform, and an isoform can, therefore, have alternative regulatory mechanisms. An example is an alternative promoter that allows transcription of the threonyl-tRNA synthetase *thrS* without a preceding T-box riboswitch [8, 11], and without the upstream genes of the operon (*dnaB*, *dnaI*, *ytxB* and *ytxC*) [4, 8]. To our knowledge, there is no *B. subtilis* annotation resource that contains a comprehensive collection of isoforms and UTRs. The lack of UTR annotation imposes a limitation on annotating and subsequently studying the function of structured RNA elements and ncRNA.

Likewise, the annotation of structured RNA elements and ncRNAs is as important as CDSs to understanding the biology of *B. subtilis *. As indicated above, structured RNA, such as riboswitches, can regulate gene expression through ligand-dependent rearrangements of their RNA structure, leading to a decreased access of the ribosome to the Shine–Dalgarno sequence [12] or to termination before transcription of the CDS. An example is the flavin mononucleotide (FMN) detecting riboswitch structure, which regulates expression of riboflavin (vitamin B2) biosynthesizing genes depending on riboflavin levels [13, 14]. More complex ncRNA regulatory interactions involve small RNAs (sRNAs) and other independently transcribed RNA structures. Some sRNAs regulate specific biological functions: for instance, the sRNA *fsrA* controls iron-metabolism [15], and the toxin–anti-toxin system of *bsrG* and *bsrE* are regulated by sRNA [16, 17]. In contrast, the 6S sRNA regulates the global transcription levels of every single gene in the genome via interaction with RNA polymerase [18–20]. Most sRNAs function by directly hybridizing to mRNA and, thereby, impact translational efficiency or mRNA stability [21]. Some ncRNAs, known as antisense RNAs (asRNAs), are expressed antisense to mRNAs and may influence the expression of the gene in the sense direction [22]. For instance, the antitoxin *ratA* regulates as asRNA the expression of *txpA* [23].

As is evident from the existing *B. subtilis* genome annotation and other relevant resources, summarized in Table 1, that the available information of ncRNAs is widely distributed and in part complementary. The genome annotation resources contain information ranging from high-quality curated annotations, over experimental data, to computational predictions. All the resources differ in their content, some provide genomic coordinates for genes, operons, UTRs and other genomic elements, while other resources contain the annotation of biological functions, gene interactions and additional meta-information. The RefSeq reference genome sequence of *B. subtilis* strain 168 [24, 25] is the standard genome relative to which most experimental studies state their gene coordinates [4, 6, 26]. This reference is also the basis for many databases and online analysis resources that use either the identical or a very similar annotation [8, 27–30]. In addition, a few ncRNA focused databases also use the RefSeq genome as reference [31–34]. Currently, a user would not only have to consult multiple of the available resources for a comprehensive annotation – which also requires the removal of redundant information – but also need user consideration of inconsistent annotation. The common use of RefSeq’s reference genome, however, makes it possible to combine the information of all these resources in a single annotation.

**Table 1.** An overview of relevant *B. subtilis* annotation resources. The first column provides the resource name. Resources marked with * were considered for integration into the BSGatlas. The remaining resources were already fully contained in at least one of the utilized resources. (Based on a trivial coordinate comparison, not shown). The second column gives a short description and the third the kind of annotations it provides.

Resource	General description	Provided annotation
RefSeq* [24]	Large collection of reference sequences including annotations Contains *B. subtilis* 168 standard annotation Most commonly used *B. subtilis* annotation resource	Coding genes All rRNAs, tRNAs of *B. subtilis * Only few other ncRNAs No mRNA transcripts
DBTBS* [27]	Collection of transcriptional binding factors Literature curated database	Operons TSSs/TTSs
BsubCyc* [35]	* B. subtilis* specific database Encyclopaedia of metabolism and pathways	Coding genes All rRNAs, tRNAs Only few other ncRNAs Few transcripts with TSSs and TTSs
SubtiWiki* [8]	* B. subtilis* specific database Active community annotation effort Functional annotation driven Does not provide coordinates	Coding genes All rRNAs, tRNAs Many additional ncRNAs TUs
Nicolas et al.* [4]	Tilling-array study in *B. subtilis * Transcriptome in over >100 conditions Large number of predictions Technical resolution limitation	Many predicted ncRNAs Many predicted TSSs/TTSs Explicitly annotates UTRs
Dar et al.* [26]	Term-seq study in *B. subtilis *	Riboswitches
Rfam* [38]	Database of RNA structure families We scanned *B. subtilis* genome with most recent Rfam version Database of RNA structure families	All rRNAs, tRNAs Riboswitches sRNAs and other structured RNAs
Ensembl [85]	* B. subtilis* specific database Active community annotation effort Functional annotation driven Does not provide coordinates	Coding genes All rRNAs, tRNAs Only few other ncRNAs No mRNA transcripts
PARTIC [29]	Integrative database of bacterial genomes Analysis tools to support biomedical research Uses different genome sequence than RefSeq But nearly identical on annotated gene set	Coding genes All rRNAs, tRNAs Only few other ncRNAs No mRNA transcripts
IMG/M [30]	Integrative database of Archaea, Bacteria and Eukarya Tools for comparative genome analysis Uses RefSeq as reference	Coding genes All rRNAs, tRNAs Only few other ncRNAs No mRNA transcripts
BSRD [33]	Single ncRNA type specific database Already included in other resources	sRNA
SRPDB [31]	Single ncRNA type specific database Already included in other resources	SRP
tmRDB [31]	Single ncRNA type specific database Already included in other resources	tmRNA
tmRNA [34]	Single ncRNA type specific database Already included in other resources	tmRNA
tRNAdb [32]	Single ncRNA type specific database Already included in other resources	tRNA

At the outset for the resources listed in Table 1, we present a combined annotation, where we focus on a set of the highest quality resources available for *B. subtilis *. These resources differ slightly in annotation terminology, but more critically they also differ in content. The database of transcriptional regulation in *B. subtilis* (DBTBS) [27] contains annotation of transcription factor binding sites, transcription start sites (TSSs), transcription termination sites (TTSs) and operons that have been extracted from hundreds of literature references. The operon annotations in DBTBS comprise the full regions of co-transcribed genes, including the possibly shorter isoforms. The annotation of DBTBS is partially contained in the high-quality curated databases BsubCyc [35], which focuses more on gene annotation, and contains an impressive amount of meta-information. However, BsubCyc provides both explicit TSSs and TTSs for only 60 % of its transcriptional annotations. Additionally, BsubCyc provides gene function in the form of Gene Ontology (GO) terms [36, 37], and transcriptional regulatory relationships, and a fine-grained, stoichiometric annotation of enzymatic reactions and molecular interactions, which are relevant for metabolomic studies. However, both DBTBS and BsubCyc are discontinued projects. In contrast, SubtiWiki [8] is a popular, active community-driven project focused on *B. subtilis* annotation [6]. SubtiWiki provides abundant meta-information, including lists of organism-specific gene categorizations, transcriptional regulations and interactions. SubtiWiki also provides a list of transcriptional annotations, but without any TSS or TTS indication, such that they only express that there exists at least one transcript that co-transcribes the genes listed in SubtiWiki’s operons. Recently, SubtiWiki included the expert gene annotation by Borriss *et al.* [6]. SubtiWiki also contains annotations of ncRNAs and UTRs, which are based on a large-scale study investigating the transcriptome of *B. subtilis* in over a hundred different environmental conditions using tiling-arrays [4]. The main strength of SubtiWiki is abundant meta-information. Unfortunately, SubtiWiki does not provide direct access to what genome coordinates that meta-information belongs to. Moreover, the above-listed resources remain protein-centric [6, 8], and predominately contain annotations of CDSs and their functions. All of these resources annotate the tRNAs and rRNAs of *B. subtilis *. Yet, these resources do not reflect the state-of-the-art information for structured RNA elements and ncRNA that can be drawn from Rfam [38]. These RNA annotations can be made available for the *B. subtilis* genome by similarity searches [39]. As demonstrated in recent work [40, 41], there is still strong potential to discover new ncRNA types and structures in bacterial genomes. An improved ncRNA annotation will lead to improved interpretation of, for example, RNA-seq data, as the ncRNA aspect is ignored in most bacterial expression analyses [3, 42, 43].

CRISPR technology is a powerful genome-editing tool and its applications are becoming fundamental also within the *B. subtilis* community [44, 45]. The CRISPR–Cas9 system forms a riboprotein complex together with a guide RNA (gRNA) molecule, which binds the first 20 nt of the gRNA to complementary DNA when the binding site is followed by the PAM sequence (for SpCas9 the PAM sequence is NGG). Several methods exist to predict the sequence-based on-target efficiency and specificity/off-targeting potential of a gRNA, such as Azimuth score [46] for efficiency and CRISPRspec score for specificity [47]. In order to achieve an intended editing, it is advised to use a gRNA sequence with high efficiency. On top of this, it is important to select the gRNAs with high specificity to minimize the potential off-targets, even though the *B. subtilis* genome is only ~4.3 million bp long.

Here, we describe the *B. subtilis* genome atlas BSGatlas, which is a coordinate-based annotation of genomic elements covering coding and non-coding genes, structured RNA, UTRs, riboswitches, TSSs, TTSs, transcripts and operons by integrating existing resources and novel genome-wide *in silico* predictions of ncRNAs. BsubCyc [35], SubtiWiki [4, 8], RefSeq [24], literature references [4, 26] and our Rfam scan [38, 39] provide a non-redundant genome annotation set, which integrates the information found in the resources listed in Table 1. Through the integration of the information from the different resources, we provide a more complete annotation of the *B. subtilis* genome, its genes, including ncRNAs, and resolve inconsistencies between the available annotations. To facilitate the use of our annotation, we furthermore provide a UCSC genome browser-based interface for convenient visualization and data download. Finally, we leveraged the operon annotations computed in this study to compute a genome-wide list of CRISPR–Cas9 gRNA that also considers applications with co-transcriptional relationships [48].

## Methods

### Computational workflow

All analyses, if not otherwise indicated, were performed in R 3.5.2 [49]. We utilized a multitude of Bioconductor packages [50] and the tidyverse collection (1.2.1) [51]. The predominately utilized packages were rtracklayer (1.42.1) [52], the annotation packages GenomicRanges (1.34.0) [53] and plyranges (1.2.0) [54], the parser genbankr (1.10.0) [55], the colour palette ggsci (2.9) [56], the library for nucleotide sequence handling Biostrings (2.50.2) [57], the graph analysis tool tidygraph (1.1.0) [58], and the table creation package kableExtra (1.1.0) [59]. For improved reproducibility of the annotation construction, all steps were conducted in an Anaconda environment. Thus, the exact list of versions for these packages and all their dependencies at each step of the annotation creation is explicitly stated. The Anaconda environment and the scripts including intermediate computational results are available at https://doi.org/10.5281/zenodo.3478329.

### Gene annotation resources

We collected annotations according to the latest RefSeq reference assembly (accession no. ASM904v1) [24, 60], which contained the major gene annotation refinement from February 2018 [6]. We used this annotation as described in the GenBank file. Based on the provided human-readable gene description text, we were able to determine the specific ncRNA type for 92 % of the 212 non-coding genes. The BsubCyc database [35] version 38 (released August 9 2017) has a systems view representation. We built a custom parser to extract for BsubCyc’s database, which contains 4188 coding and 184 non-coding genes and RNA structures. Also, BsubCyc’s curation contained experimentally verified information about 574 TSSs with nucleotide resolution and 1246 TTSs. These provide clear transcribed boundaries for some of the 1602 transcriptional units (TUs) from BsubCyc, yet explicit UTR elements were not annotated. As meta-information, BsubCyc provides GO terms and indication of transcriptional regulatory relationships. In addition, BsubCyc gives a detailed metabolic and enzymatic reaction and pathway overview, which will not be considered here.

We included the annotations by Nicolas *et al.* [4] of 3242 TSSs, 2126 TTSs, 1430 UTRs and 153 novel ncRNA. Dar *et al.* [26] found a set of 82 riboswitches that they found by investigating transcription termination patterns in a Term-seq experiment. The other annotation resources required more dedicated pre-processing, see below.

### Parsing DBTBS

Upon request, the authors of DBTBS [27] kindly provided us with their latest annotation in an XML format (version 2008). We added coordinates to their information with an exact sequence lookup to find coordinates for 98 % of the 1*2*62 annotated transcription factor binding sites and for 90 % of the 1031 annotated TTSs. To reduce erroneous or ambiguous annotations, we used unique matches without allowed mismatches. From the sigma factor binding sites that have the relative position to the TSS provided, we were able to infer a set of 644 high-resolution TSS positions. DBTBS had a curated annotation of operons for 2*2*01 genes. For 98.9 % of these genes, we were able to find the corresponding gene in our merged set by comparing the gene names and keeping only unambiguous matches. Due to the high matching success rate of the genes, we were able to fully restore coordinates for almost all (98.6 %) of the annotated 1123 operons.

### Parsing SubtiWiki

SubtiWiki provided a magnitude of meta-information, such as its gene categorization and lists of transcriptional regulations and interactions [8]. In the most recent version (downloaded November 9 2020) SubtiWiki included some parts of BsubCyc [8, 35], yet the curated list of TSSs, TTSs and functional annotation via GO terms were not included. Unfortunately, SubtiWiki does not provide the export of gene coordinates, such that we restored these from our merged gene set via comparison of loci, gene names and synonyms. We were able to infer positions for 99.7 % of the 5999 coding/non-coding genes, structures and UTRs described in SubtiWiki; for 99.6 % of the 2*2*67 provided SubtiWiki TUs.

### Rfam scan

The genome sequence was scanned for the 3016 families of Rfam 14.1 [38] using Infernal’s cmsearch version 1.1.1 [39]. The scan was conducted with an *E* value cut-off of 10^–3^, which in most cases is more relaxed than the family-specific so-called gathering score (the 16 families where the gathering score cut-off would have been a more relaxed cut-off are not expected to be present in bacteria). The hits from the scan are reported here at three confidence levels [1]. At the *conservative level*, an *E* value cut-off at 10^–6^ and a match score of at least the gathering score was applied [2]; at the *medium level*, only the *E* value cut-off at 10^–6^ was used; and [3] at the *relaxed confidence level*, the *E* value requirement was relaxed to 10^–3^. The results were post-filtered using an updated version of the RNAnnotator pipeline [61]. Within each level of confidence, all hits overlapping at least 40 bp were merged and the best hit by *E* value was chosen (or by score if *E* values were tied). At the respective confidence levels, the scan identified 214, 230 and 285 non-coding candidate genes and structures, respectively. We did not filter the Rfam collection before the scan, such that apparent false-positive cases are included. After inspection of the scans, these false-positives indicated that the most relaxed level of Rfam should be excluded due to the erroneous prediction of matches to ncRNA families not expected in bacteria. The results of the relaxed scan are still shown in the browser as they could prove interesting after manual curation.

### Merging of gene annotations

First, for each overlapping pair of gene annotations, we computed the ratio of lengths of the intersection and the union, which can also be recognized as the Jaccard index (JI) [62]. Then, we determined a cut-off in JI to identify annotations corresponding to each other by first considering their identifiers (name or locus tag). For corresponding coding genes, we observed that the JI was at least 0.80, while for ncRNAs it was at least 0.5, except when one ncRNA annotation was fully contained within another. Using these JI cut-offs, we inferred correspondence in the remaining pairs of overlapping annotations of coding genes, *de novo* ncRNAs and structured RNAs. Overlaps between coding genes and riboswitches are, however, excluded. Accordingly, we merged overlapping annotations into a single annotation with the coordinates of the highest priority resource (if there were multiple highest priority annotations, the union of the coordinates was used; Fig. S1a, available with the online version of this article). For the prioritization of the resources see Table S1. The pseudo-code of the merging is outlined in Fig. S2. The merged annotations were assigned the most specific biotype (e.g. sRNA instead of putative non-coding) from the individual resources. In a few cases, we resolved ambiguities by preferring the biotype asRNA over sRNA, and sRNA over riboswitch. The latter ambiguity is caused by the Term-seq resource [26].

### Transcriptional Units

Here, and in contrast to others, for simplicity we use the term transcriptional unit (TU) as a set of genes that can be transcribed together, without precise indication of transcript boundaries. Hence, the TU can refer to one or more transcripts covering an operon. Due to more complex operons, multiple transcripts can refer to the same TU (see Operon architecture). We collected a set of 1602 TUs from BsubCyc [35], 2483 TUs from SubtiWiki [8] and 1123 TUs that are implied by operons from DBTBS [27]. We complemented these TUs with the merged genes if (a) a gene was fully contained by the TU, (b) if it contained the TU or (c) the overlap relative to the length of the gene was at least 70 %. Overall, we added genes to 46 TUs from BsubCyc, 753 TUs of DBTBS and 1968 TUs from SubtiWiki. We removed two TUs, one from DBTBS and SubtiWiki each, which were erroneous as they would span more than a quarter of the whole genome. The resulting set of unified TUs totalled 2483 TUs.

### Transcription start and terminaton sites

We unified existing annotations of promoters and their TSSs and TTSs. We followed, for each type separately, an approach similar to the gene merging step. We first compared the distances and overlaps between the annotation to determine their resolution limits (Fig. S3). We determined for each data type the resolution limits (see Results). Assuming single-nucleotide resolution for BsubCyc and DBTBS, and ±22 bp limits for Nicolas *et al.* predictions (two times the tiling-array interval), we merged the TSSs and TTSs separately in an approach similar to the gene merging. Instead of a resource priority, we resolved each overlapping group by keeping those annotations with the best resolutions. For each merged set of TSSs, we collected the information on which sigma factor promotes the transcription from the entry with the lowest resolution limit.

### Untranslated regions

Within a distance cut-off of 2000 bp, we created a TSS and TTS map by associating TSS to the gene with the closest 5′ end and TTS to the one with the closest 3′ end (Figs S4 and S1b). We lowered the cut-off to 200 bp for the Nicolas *et al.* [4] annotations that had no previously associated transcriptional region. If there was a gap between a TTS and the associated gene of at least 15 bp (due to the resolution limits), we created a 3′ UTR in its place. We similarly created 5′ UTRs based on TSSs. However, we extended the length of 5′ UTRs up to the next coding gene if the first direct association is a ncRNA structure. We filled gaps longer than 15 bp between the genes listed in the TU with internal UTRs. The only exception is that we did not add an internal UTR for the *sigK* TU, because the over 10 000 region within it is not actually transcribed due to its unique regulatory mechanism [63].

### Operon architecture

The genes of one TU can be transcribed by one or multiple transcripts (see Transcriptional units). Due to isoform transcripts, the computation of operons needs to consider transcripts/TU that do not contain all genes, similar to how DBTBS annotates operons. We compute operons based on the TSS, TTS, UTR, and gene annotations. We also inferred novel TUs and the full transcripts by detecting paths in a directed graph (Fig. S1c). The nodes are TSSs, TTSs, UTRs and genes. We added dummy TSSs and TTSs for TUs whose genes had none associated. We connected the nodes with directed edges in the direction of transcription (Fig. S1c), e.g. a TSS is connected to the 5′ UTR it is associated with and from there to a coding gene. Each path that connects TSS and TTS represents a transcript that contains the genes and UTRs along each path, and TUs when only considering genes. Subsequently, we computed the operon architecture of the transcripts and genes in a second graph, which had genes and transcripts as nodes and edges between transcripts and the associated genes. The operons are represented by the connected components in the graph (Fig. S1d). Due to the particularity of the *sigK* transcriptional regulation [63], we excluded the associated TU both from the transcript and operon inference.

### Browser hub

We generated the browser hub according to the official UCSC genome browser documentation and converted the tracks into the custom binary format with the UCSC tools. The individual tracks were organized according to the track definition (https://genome.ucsc.edu/goldenPath/help/trackDb/trackDbHub.html) and the search functionality was provided as a Trix index (https://genome.ucsc.edu/goldenPath/help/trix.html). We created a color-blind friendly color scheme to indicate the biotype in the browser (Fig. S5).

### Inclusion of tiling array signals

The tiling-array was designed for an older reference genome sequence (accession no. NC_000964.2). We transferred the information to the most recent reference sequence (NC_000964.3) with UCSC genome browser tool liftOver (version 377) based on a pairwise genome alignment generated with lastz (version 1.04.00) [64, 65]. We extracted the log2 transformed foreground signals for the entire dataset (GSE27219). Instead of a single-nucleotide probe position, we show in the browser the coverage signal extended up to the starting position of the next probe (~22 nt). We also added information for the full probe oligonucleotides (45–65 nt). In the liftOver, we only allowed transfer for exact matches (minMatch=1). We discarded probes that would have changed the strand after lifting. In total, 2419 of 383 143 (0.6 %) probes could not be lifted over. Yet, the tiling intervals after lifting were still 22 nt for 99.7 % of probes. Nicolas *et al.* [4] conducted an additional normalization step of the signals; however, the tool they used is no longer available. Given that the study that designed the array suggested that no extra normalization might be needed [66], we did not conduct a normalization of the signals.

### CRISPR guide predictions

We predicted gRNAs binding to the *B. subtilis* genome and scored their specificity as CRISPR–Cas9 guides with CRISPRoff pipeline (v1.1) and RIsearch2 (v2.1) [47, 67]. For the computations, we used the default setting for both tools as recommended. Potential off-targets with up to six mismatches were considered for the estimation of specificity. For all identified on-targets, their efficiency was estimated e.g. with the Azimuth (v2.0) score [46]. The predicted cut-positions of all on-/off-targets were checked for overlaps with BSGatlas with the tools listed under ‘Computational workflow’.

## Results and Discussion

### Enhanced annotation of coding genes, and non-coding and structured RNAs

To obtain an enhanced annotation of the genomic coordinates of coding and non-coding genes, we collected resource-specific gene annotations for *B. subtilis* strain 168 from BsubCyc [35], RefSeq [6, 24, 60], SubtiWiki, the tiling-array based predictions by Nicolas *et al.* [4, 8] and the riboswitches identified by Dar *et al.* [26] (Table 1). In addition to these resources, we included our computational screen (see Methods) of the *B. subtilis* genome for non-coding and structured RNAs using Infernal and Rfam version 14.1 [38, 39]. We generated a combined gene annotation set that addresses both redundancies and discrepancies between and within the resources. We first prioritized the resources according to prior knowledge about their reliability, e.g. manually curated resources are more trustworthy than computational predictions (Table S1). Afterward, we found and inspected all pairs of gene annotations with overlaps in gene coordinates and employed tailored strategies for how these should be resolved. In general, the annotation of a higher prioritized resource will replace an overlapping annotation of lower priority if they fulfil the criterion for matching gene pairs, as described below, to prevent mixing of annotations, e.g. of coding genes and RNA structures. Finally, we integrated the meta-information from the individual resources into the merged gene set.

*Rfam screen.* We scanned the *B. subtilis* genome with the entire Rfam model collection, including models for non-bacterial RNAs, as a control to tune the prediction parameters, Infernal’s scores and *E* values according to our chosen annotation confidence levels: conservative, medium and relaxed (see Methods). Seven non-bacterial hits were found for the relaxed confidence level (*E* value <10^−3^, no score cut-off): four miRNA families (MIR159, MIR167_1, mir-580 and mir-598), *RUF1* from eukaryotes and two families from archaea (*sRNA162* and the CD-box snoRNA *sR2*). These are more hits than the single hit that would be expected by chance (1066 non-bacterial families and *E* value cut-off of 10^−3^). In contrast, less than one random hit should be expected for an *E* value cut-off of 10^−6^, which was used for the conservative and medium confidence levels. And indeed, all hits from the conservative and medium confidence levels were from bacterial families only. The conservative confidence level had 214 Rfam in the *B. subtilis* genome of which 17 (8 %) were not in any previous annotation resource dedicated to *B. subtilis* (as determined after gene merging). These 17 annotations were 8 Bacillaceae-1 structures, 1 *T-box* riboswitch, 1 *cspA* thermoregulator, 3 presumed cis-regulatory structures (*yjdF*, *epsC* and *PyrG*-leader), the asRNA structure *dicF,* a sRNA described in some bacterial *ureB* 5′ UTRs (although our Rfam scan located the structure anti-sense downstream of *B. subtilis ureB)*, an additional toxin–anti-toxin sRNA *bsrC* (between the coding genes *cspC* and *ydeB*), and a sRNA structure usually associated with bacterial plasmids (between *nicK* and *ydcS)*. An additional 16 matches were obtained at the medium confidence level, 8 of these were not annotated by any previous annotation resource (see below). These eight ncRNA hits were two *cspA* RNA thermometers structures [68], the asRNAs *rliD* [69] and *AsrC* [70], the sRNA *bsrG* [16], *rli54* [71], *Ysr141* [72] and an additional self-splicing intron [73] within the ribonucleoside-diphosphate reductase gene *nrdFB.* The relaxed level adds 48 more candidates than the medium confidence level (excluding the 7 non-bacterial hits). Given the non-bacterial hits, we excluded the relaxed scan from the merging, yet we provide it as supplementary information for more putative ncRNA candidates.

*Gene annotation resources and prioritization.* The BsubCyc annotation contains 4188 coding, and 183 non-coding genes and structured RNA annotations. RefSeq contains a similar number (4325 coding and 212 non-coding genes). SubtiWiki provides meta-information for the genes listed by RefSeq and for the 153 transcribed regions predicted to be non-coding by Nicolas *et al.* (excluding predicted UTR regions) [4]. Dar *et al.* predict 82 regions as potential riboswitches [26]. In Table S1, we list the individual priorities of the resources we used in the merging procedure. Instead of an iterative merging procedure that might depend on the order of merging, we use a single step merging (Fig. S2, cut-offs derived in the next paragraph). We prioritize RefSeq over BsubCyc, because it contains the most recent expert curation of the *B. subtilis* genome coordinates [6]. To prevent that ncRNA annotation from RefSeq conflict with coding annotations from BsubCyc, we furthermore split protein-encoding genes and ncRNAs into separate sub-resources and prioritized them separately. Following the distribution of annotation similarities (Fig. S6), we consider ncRNA information from the Rfam screen to be of equal quality as those from RefSeq and BsubCyc (due to highly similar annotations; Fig. S6). Thus, we ranked Rfam and RefSeq equally; however, to allow RefSeq as the newer reference annotation to overwrite BsubCyc, we ranked BsubCyc lower. The riboswitch predictions by Dar *et al.* are based on Term-seq experiments, such that these should be more accurate than tiling-array-based annotations. Therefore, we rank these higher than the Nicolas *et al.* predictions [4]. Given the proposed high-resolution potential of the Dar *et al.* predictions, we prioritized them equally with the non-coding part of BsubCyc. We ranked the medium confidence Rfam scan equally with the Nicolas *et al.* predictions. In short, we prioritized according to the principle of coding over non-coding resources, manually curated annotations over *in silico* generated annotations and high over low resolution.

*Matching of overlapping gene pairs from two resources.* To decide when overlapping annotation pairs from two different resources should be merged, we calculated the ratio of lengths of the intersection and the union, which can also be recognized as the JIs, and inspected the distributions of JIs both between and within resources (Fig. S6). These showed that nearly all overlapping CDS annotations were either very similar (JI *>*0.98) or very dissimilar (JI *<*0.1). The dissimilar cases were at least in part due to the on-going curation efforts [6]. In general, CDSs do not overlap ncRNAs with the exception of riboswitches and a single overlap with a predicted ncRNA from a low confidence resource (S1078). For the overlaps between ncRNAs, the JI distributions depend on the resource. In particular the ncRNA predictions from the tilling-array study [4] tended to be less similar to the annotations from manually curated resources (predominately JI >0.8, but even as low as JI ~0.5 when comparing to manually curated resources), which is due to the constrained resolution of the underlying technology employed by Nicolas *et al.* The instances of very low JIs of some non-coding annotations in comparison to coding annotations was due to their short length (see also subsection ‘Improvements in gene coordinates’). For example, Dar *et al.* [26] predicted a 182 bp long riboswitch upstream of *yxjH* that overlaps (107 bp, JI 0.59) with a SAM riboswitch annotation from RefSeq (BSU_misc_RNA_61). We also investigated the distribution of JI for gene pairs that corresponded to each other by matching gene names and matching locus tags. We found that all such corresponding genes, both coding and non-coding, had a JI above 0.8, with the exceptions of two different annotations for the spore coat protein *cotT* (JI=0.77), *yoyG* (JI=0.49), *yqjU* (JI=0.53) and *yrzH* (JI=0.45). Therefore, we decided to keep both annotations for these four genes as separate entries. These gene annotation differences cannot be resolved by homology, according to their BsuCyc entries.

*Gene merging.* In conclusion of the last paragraph, we merged overlapping genes if one of the following criteria was met: (i) their JI was at least 0.8, (ii) both annotations were non-coding and their JI was at least 0.5, or (iii) a ncRNA annotation was fully contained within another. However, riboswitches were not merged with overlapping coding genes. For the merged annotation, we used the coordinates from the resource with the highest priority, or the union of the overlapping coordinates if multiple resources had the highest priority (Fig. S1a). In practice, the latter only applied to the ncRNA resources. The merging procedure is outlined as pseudo-code in Fig. S2. In total, the merging generated a non-redundant set of 4332 coding genes and 441 ncRNAs, which included 103 cis-regulatory RNA structure elements. We assigned each merged gene the most specific biotype found in the group (e.g. sRNA instead of putative predicted ncRNA). Finally, we checked locus tags and gene names and found that there were no erroneously merged genes or missed gene pairs. Note that in this approach we merged across all priority levels and at the same time merged annotations within the same level, possibly even within the same resource (Fig. S1a).

*Association of meta-information.* By traversing the merging steps backward, a comprehensive set of meta-information can be associated with the merged gene annotations. This includes general descriptive texts, synonyms, molecular masses of translated proteins and literature references. By using the locus tags provided from the individual resources, we looked up the corresponding entry SubtiWiki and included its meta-information. We preferentially used the primary naming used in SubtiWiki. In addition, we added BsubCyc’s functional annotation with GO terms [36, 37] for 71 % of all genes and enzyme classifications from SubtiWiki (14.5 % of all genes), RefSeq (19.5 %) and BsubCyc (15.7 %), which in combination covers nearly 22 % of all genes. We recoded these enzyme classifications into a human-readable format according to the definitions in the BRENDA database [74]. Finally, we added the information available in SubtiWiki’s *B. subtilis* specific category system, which contained information about 91.9 % of genes. We also associated KEGG pathway information with 28.6 % of genes via using KEGG’s REST interface [75]. From the Nicolas *et al.* tilling-array study [4], we listed experimental conditions with the highest and lowest expression and genes with a correlated expression as they provided. We did not further filter the meta-information, but for each piece of information, we indicated its origin.

*Improvements in the number of ncRNA annotations.* In comparison to the latest RefSeq assembly, our merged gene annotation contained 196 additional ncRNA genes and structured RNA elements (Table 2), of which 64 ncRNAs have a clear annotation of the ncRNA biotype (e.g. sRNA instead of putative ncRNA). A total of 27 of these 64 ncRNA were exclusively based on Dar *et al.* [26] riboswitches and 25 were exclusively from Rfam hits. Only one of the remaining 12 had no overlap with an Rfam hit; it originated from BsubCyc and a Nicolas *et al.* prediction. Seven were Nicolas *et al.* predictions [4] (with Rfam hit). The rest were Dar *et al.* detected riboswitches, of which two had an overlapping Nicolas *et al.* prediction. The remaining 132 ncRNAs of less specific non-coding biotype originated from the Nicolas *et al.* predictions. In total, the number of annotated known sRNAs, asRNAs and riboswitches doubled in the merged gene set in comparison to the RefSeq annotation.

**Table 2.** Comparison in number of annotations after merging. The number of annotations are shown for the individual resources (columns 3–7) in comparison to the merged results (column 2). For purposes of readability, the conservative and medium Rfam confidence levels have been aggregated, as well as the coding/non-coding parts of RefSeq and BsubCyc. The full, unaggregated table is shown in Table S1. The table below is subdivided into coding (first four rows of data) and non-coding annotations (remaining rows of data). Both the coding and the non-coding parts start with a row showing the total number of annotations. The lines below these rows further qualify these with respect to potential putative annotation status or a specific non-coding biotype. The percentages are relative to the number in each block.

Gene annotation	Merged result	Individual resources				
		RefSeq	BsubCyc	Dar *et al.* riboswitches [26]	Rfam	Nicolas *et al.* predictions [4]
Protein-encoding genes	4332	4324	4188	–	–	–
Putative/predictions	79 (2 %)	88 (2 %)	1210 (29 %)	–	–	–
Hypothetical status removed	–	9 (0.2 %)	1204 (29 %)	–	–	–
Resource specific genes	–	144 (3 %)	8 (0.2 %)	–	–	–
ncRNAs	408	212	183	82	230	153
rRNA	30 (7 %)	30 (14 %)	30 (16 %)	0 (0 %)	30 (13 %)	0 (0 %)
tRNA	86 (21 %)	86 (41 %)	86 (47 %)	0 (0 %)	86 (38 %)	0 (0 %)
Small regulatory RNA	37 (9 %)	14 (7 %)	9 (5 %)	0 (0 %)	31 (13 %)	0 (0 %)
Regulatory asRNA	8 (2 %)	3 (1 %)	2 (1 %)	0 (0 %)	4 (1 %)	0 (0 %)
Riboswitch	104 (25 %)	55 (26 %)	26 (14 %)	82 (100 %)	73 (32 %)	0 (0 %)
Self-splicing intron	3 (1 %)	0 (0 %)	0 (0 %)	0 (0 %)	3 (1 %)	0 (0 %)
Ribozyme, SRP, tmRNA	3 (1 %)	2 (1 %)	2 (1 %)	0 (0 %)	3 (1 %)	0 (0 %)
Putative/predictions	137 (34 %)	22 (10 %)	28 (15 %)	0 (0 %)	0 (0 %)	153 (100 %)
Hypothetical status removed	–	17 (8 %)	29 (16 %)	0 (0 %)	0 (0 %)	19 (12 %)
Reclassified as coding	–	–	–	–	–	1 (0.7 %)
Resource-specific genes	–	10 (5 %)	–	27 (33 %)	25 (11 %)	133 (87 %)

*Improvements in gene coordinates.* We inspected refinements in coordinates by the difference in the number of bp (Table S2). Unless explicitly mentioned otherwise, we use bp in the context of genomic coordinates. Not surprisingly, we find that the ncRNAs show a lower JI for a given bp difference simply because in general they are shorter than the protein-encoding genes (Fig. S7). Because RefSeq’s coding genes are assigned the highest priority in the merging step, no coordinates changed for them with the exception of an artefact, a predicted ORF (RefSeq entry BSU36079, comment ‘doubtful CDS’), which was merged into the larger coding gene *cotG* containing it. We observe that 49 coding genes annotated in BsubCyc has coordinate changes between 10 and 250 bp, although, due to the lengths of the genes, the overall JI, between the respective original annotations and the merged gene annotations, was barely reduced. In the comparison of overlaps between resources, we mentioned four highly divergently annotated CDSs with low JI between RefSeq and BsubCyc. Besides these, BsubCyc only uniquely annotated three more hypothetical proteins (*yfmA*, *ypzE* and *ytzK*). Otherwise, all coding genes of BsubCyc were found in RefSeq. Thus, our merging had a limited effect on CDS coordinates, likely reflecting the high quality of the protein annotation. In contrast, the coordinates of 68 % of RefSeq’s and 79 % of BsubCyc’s annotated non-coding genes and structured RNA elements were refined up to 500 bp, although even smaller changes of 50 to 100 bp reduced the JI down to 0.6. These changes of BsubCyc’s and RefSeq’s non-coding annotation were the result of the merging with higher confidence resources from the literature-based resources. Moreover, two putative ncRNA from the Nicolas *et al.* [4] tiling-array study were reannotated as part of the three-component toxin–antitoxin-antitoxin system *SpoIISABC* (BSU12815, S458) and as a separate hypothetical short peptide (BSU28509, S1078).

### Promoter map and complex operon architecture

From the annotation resources stated above, only BsubCyc [35] explicitly annotates a few complete transcripts. More commonly, the transcript boundaries are not indicated (Table 1). Using our definition of TUs (see Transcriptional Units), we refer to an operon as a region of one or more genes covered by at least one common transcript or multiple transcripts covering at least one full gene sequence (both CDS and non-coding). The full boundary relative to a TU is given by an upstream TSS and a downstream TTS. In the case of isoforms of varying 5′ or 3′ UTR lengths, a TU might have multiple TSSs/TTSs. The annotations of TUs, TSSs and TTSs are stored in multiple resources, and these resources have varying accuracies in the annotations (see below); thus, the task of first combining the individual resources and afterward inferring transcripts is not straight forward. Here, we addressed this by (i) combining known TU annotations, (ii) creating a single promoter map from existing TSS annotations, (iii) integrating known TTS positions, (iv) identifying UTR elements from the location of TSS/TTS relative to TUs, and finally (v) computing individual transcripts, including isoforms of bacterial operons. However, two complicating factors needed to be considered. First, the resources do not annotate the TUs relative to the same genome sequence or might have potentially ambiguous gene names. Thus, we transferred them to the same, above merged, gene set, and complemented them accordingly with new annotations. Second, TSSs and TTSs could give rise to novel TU annotations when a TSS/TTS is located inside the region implied by a TU without an additional adjacently located upstream/downstream TU (Fig. S1c). At the end of this section, we also verified the consistency of our annotations, those of the UTRs in particular, by (i) comparing the biotypes with overlapping Nicolas *et al.* predictions [4], and (ii) checking if the annotations are reliable with the transcription signals from the tiling-array study.

*Known TUs.* BsubCyc’s transcript annotations were based on 1602 TUs, SubtiWiki stated 2267 TUs [8] and DBTBS’s operons implied 1123 TUs [27]. We investigated the overlaps of these TUs with the merged gene set and found two main overlap scenarios: either genes were fully contained by a TU, or a gene or structure had a large overlap with a TU, possibly to the extent that it fully contained it. The latter was the case for a small peptide sequence of unknown function (BSU32719) that was fully contained in a known small regulatory RNA (bsrI). Thus, we decided to complement a TU by a gene or structure if (a) the gene was fully contained by the TU, (b) if it contained the TU or (c) the overlap relative to the length of the gene was at least 70 %. The complemented, non-redundant union of these sets described 2473 TUs. Following this, we excluded the *sigK*-related TU. Its full 10 000 bp region is, due to its unique regulatory DNA excision mechanism, not actually transcribed [63].

**Fig. 1.**
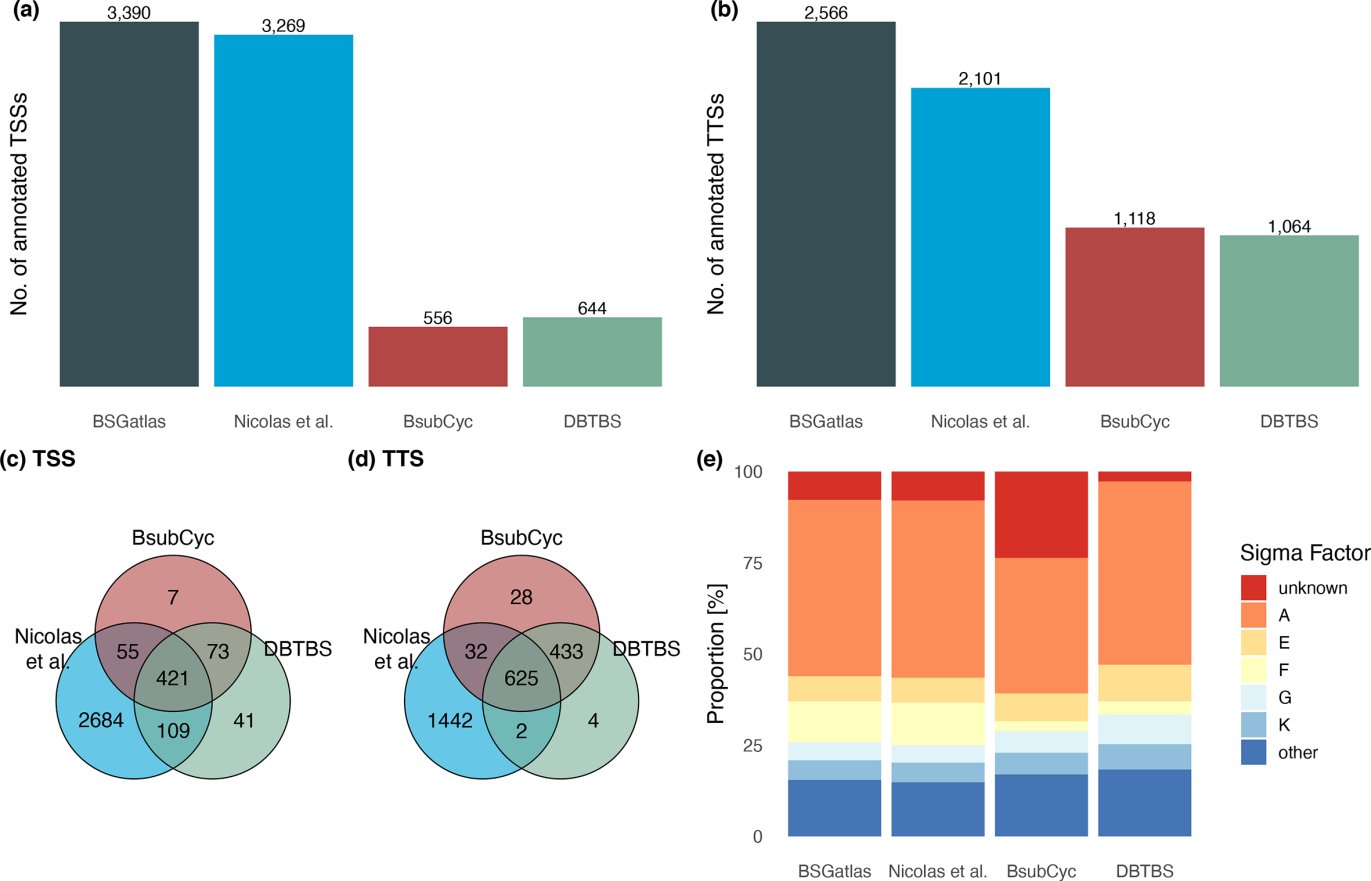
Comparing BSGatlas to the individual resources. (a) Number of TSSs and (b) TTSs. (c) Shared TSSs and (d) TTSs between the resources. (e) Distributions of sigma factor binding sites.

*Promoter and termination map.* We collected the TSS and TTS annotations from the external resources. In accordance with experimental verification stated in the descriptions, we take the BsubCyc and DBTBS TSSs as the gold standard. Indeed, these TSSs/TTSs had nucleotide resolution (Fig. S3a), which is given by the minimal distance between two separate annotations within a single resource (similar to the spatial resolution of an eye to distinguish two points). In contrast, the TSS/TTS annotations of Nicolas *et al.* had at least 45 bp between them, which is given by a window of the annotation position ±22 bp. The 22 bp is due to the tiling intervals of the tiling-array design [4, 66]. Although Nicolas *et al.* reported that 85 % of a reference set they created was within 12 bp distance to their predicted TSSs, our observations suggest that the distance to the actual curated TSS is about twice that (Fig. S3b). That increase in observed distance is due to the nature of the reference set. Nicolas *et al.* used the promoter binding sites from DBTBS, which are intervals that stretch −50 to +15 relative to the TSS. Moreover, the TSS/TTS positions of Nicolas *et al.* were predicted from a mean signal from multiple probes. Therefore, we assumed in the following that the Nicolas *et al.* TSS/TTS annotations have a resolution in a 45 bp window, in which the biological true position should be expected. Similar to the gene merging procedure, we first identified groups of TSSs (and TTSs separately) that overlapped within their resolution limits, and afterward retrieved a unified TSS and TTS map by taking per group the TSS/TTS annotations that had a resolution equal to the minimal resolution within each group. The unified map contained 3390 TSSs and 2566 TTSs (Fig. 1). The underlying annotation resources only shared a set of 12.4 % of the TSSs and 24.3 % of the TTSs. The largest quantity of annotation originated from the Nicolas *et al.* tiling-array study [4]. In the combined set, 79.2 % of the TSSs and 56.2 % of the TTSs were solely provided by the tiling-array data. In contrast, the union of the higher resolution resources BsubCyc and DBTBS provided unique annotations for 3.6 % of TSSs and 18.1 % of TTSs. Overall, a total of 20.8 % of the TSSs and 43.8 % of TTSs had both single-nucleotide resolution and an expert curation origin. In comparison, the distribution of sigma factor binding sites associated with the TSSs remained similar between the resources, although not as many promoters had an unknown sigma factor as in BsubCyc. Rapid amplification of cDNA ends (RACE) experiments might be able to add additional high-resolution TSS annotations; however, we are unaware of any studies that provide such whole-genome information for *B. subtilis* [76]. However, a small-scale RACE experiment that found five TSSs was conducted in the study that designed the array used by Nicolas *et al.* [66]. Four of these five TSSs are also contained in the TSS map computed here. The fifth (near S953) had no TSS annotation from any of our used resources. Given the small scale of the RACE experiments, we did not include them in our annotation generation; however, the comparison with that one study should illustrate that our TSS unification step does indeed provide reliable TSS annotations, in particular because only prior annotation has been used.

*UTR annotations.* The UTRs annotated by Nicolas *et al.* [4] are divided into non-overlapping pieces. Thus, these annotations only reflect the entire biological UTR regions when strung together. The total lengths of these biological 5′ and 3′ UTRs in *B. subtilis* are at most 2000 bp long, and indeed most (>95 %) of TSSs/TTSs are within that distance to the 5′/3′ end of gene annotations (Table S3, Fig. S4b). (These lengths also include potentially overlapping regulatory structures.) Moreover, 85 % of TSS annotations are located within 500 bp upstream of a gene (Fig. S4a). Although only 60 % of TTSs are clearly located downstream of a gene, the proportion is also >85 % when considering a small 25 bp overlap to account for resolution limitations and the fact that terminators are regions that fold to a hairpin structure. However, Nicolas *et al.* predicted 242 TSSs and 1125 TTSs that were not associated with a transcribed region. These were considerably further away >200 bp from gene annotations (45 % of TSSs and 25 % of TTSs; Fig. S4b). Therefore, we predicted the regions from TSSs to downstream genes and TTSs to upstream genes, within the 2000 bp distance cut-off, represent 5′ UTRs and 3′ UTRs, respectively. For those TSSs/TTSs without detected transcribed regions, we lowered the cut-off to 200 bp. After filtering for a minimum of 15 nt long UTRs, we derived a map of 2761 5′ UTRs and 1449 3′ UTRs. This approach associated 103 TSSs to downstream cis-regulatory RNA structures. In order to fully represent the UTR, we extended the predicted 5′ UTR annotation to the following coding gene. Finally, we inferred 1125 internal UTRs from the regions between genes that are known to be co-transcribed according to the above-described combined TU list. Although some of these internal UTRs overlap with what Nicolas *et al.* described as intergenic regions (Table S3), we find them ambiguous: the definition of an intergenic region is only the region between two genes; as defined by the term SO:0000605 of the sequence ontology [9]. However, intergenic regions do not distinguish 5′, 3′ or internal UTR. For all UTRs longer than 46 bp (shortest UTR in the work of Nicolas *et al.*), the distribution of our *in silico* obtained UTRs lengths were similar to those of the ones obtained by Nicolas *et al. (p_i_ <*1.2×10^–7^, two-sample Kolmogorov–Smirnov test for all UTR types *i*) (Fig. S4b). We found 2*2*16 short UTRs between 15 and 45 bp. Overall, our combination of TSS/TTS allowed for annotation of nearly five times more 5′ and 3′ UTRs than Nicolas *et al.* (4210 vs 925) and more than twice as many internal UTRs (1125 vs 505, including their intergenic regions).

*Identification of transcripts and operons.* We used the associations found between TSSs, TTSs, UTRs and genes with respect to TUs to compute transcripts (Fig. S1c). By considering TSSs or TTSs that were inside of annotated TUs, we inferred 717 novel TUs. In total, we found 4*5*17 transcripts. The number of transcripts is larger than those of TUs, because a TU can have multiple transcripts with varying UTR lengths. In comparison, BsubCyc annotated 1*6*02 transcripts. Under the assumption that two transcripts that share the full sequence of at least one gene are isoforms from the same operon, we found a set of 2*2*66 operons (Fig. S1d). In accordance with the expectation, almost all of these operons (99.7 %) had a transcript containing all of the operon’s genes. Afterward, we looked up for each transcript which TU annotation it was based on, and from which resource the TU originated. Subsequently, we computed the proportion of genes for which each resource provided transcriptional annotation. The combined set of TUs explained transcripts for 85 % of the genes, which is 3 % more than the largest individual resource. When including our novel TUs, 92 % of all genes had a transcript annotation.

**Fig. 2.**
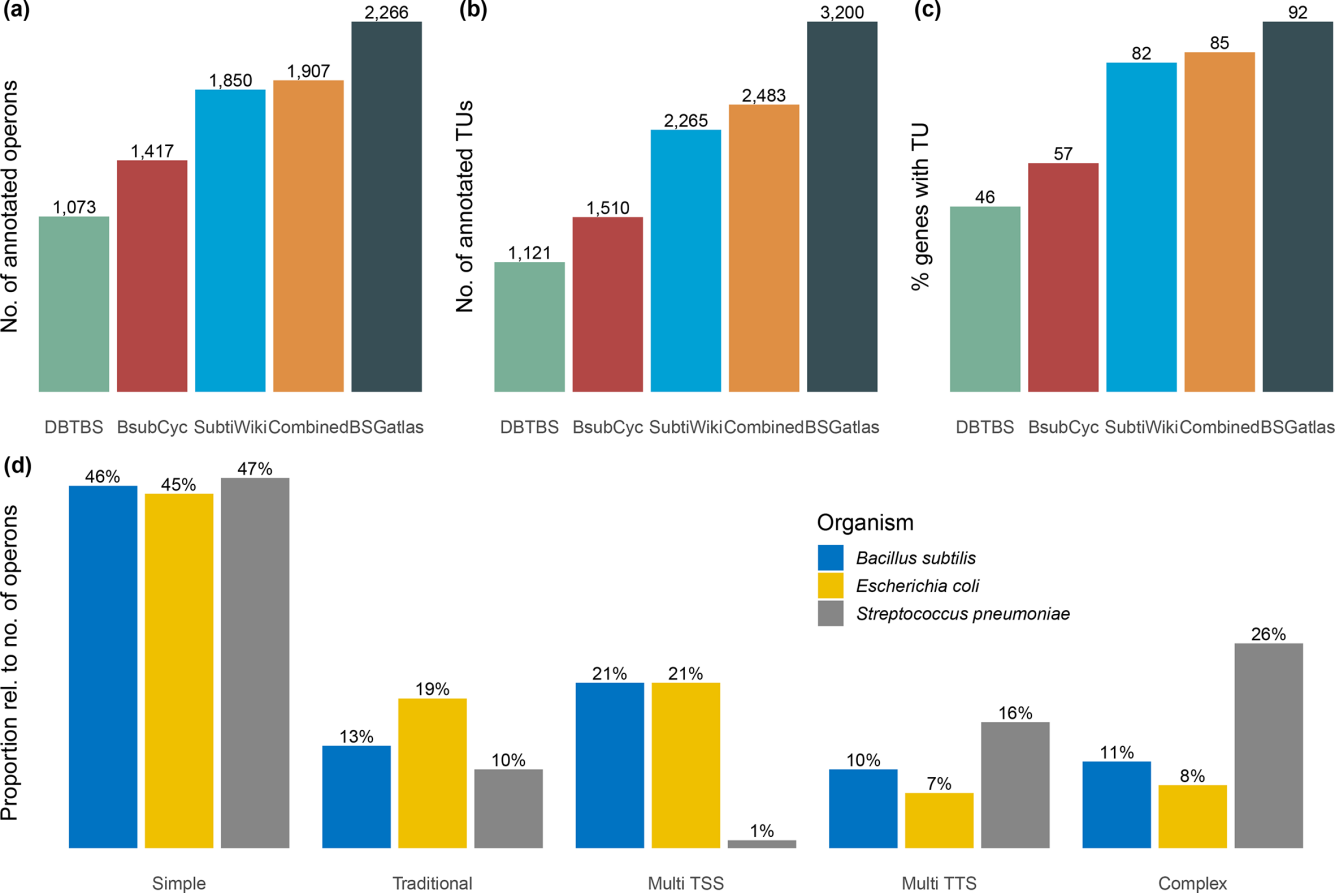
Comparing the different resources with respect to the number of (a) operons, (b) TUs and (c) the proportion of the merged gene set they cover. Here, the numbers in the ‘combined’ case (orange bars) refer to the annotation before computing novel TUs. (d) Comparison of the operon classes for our computed operons in *B. subtilis* with those found in *S. pneumoniae* and *E. coli* [10, 77].

**Fig. 3.**
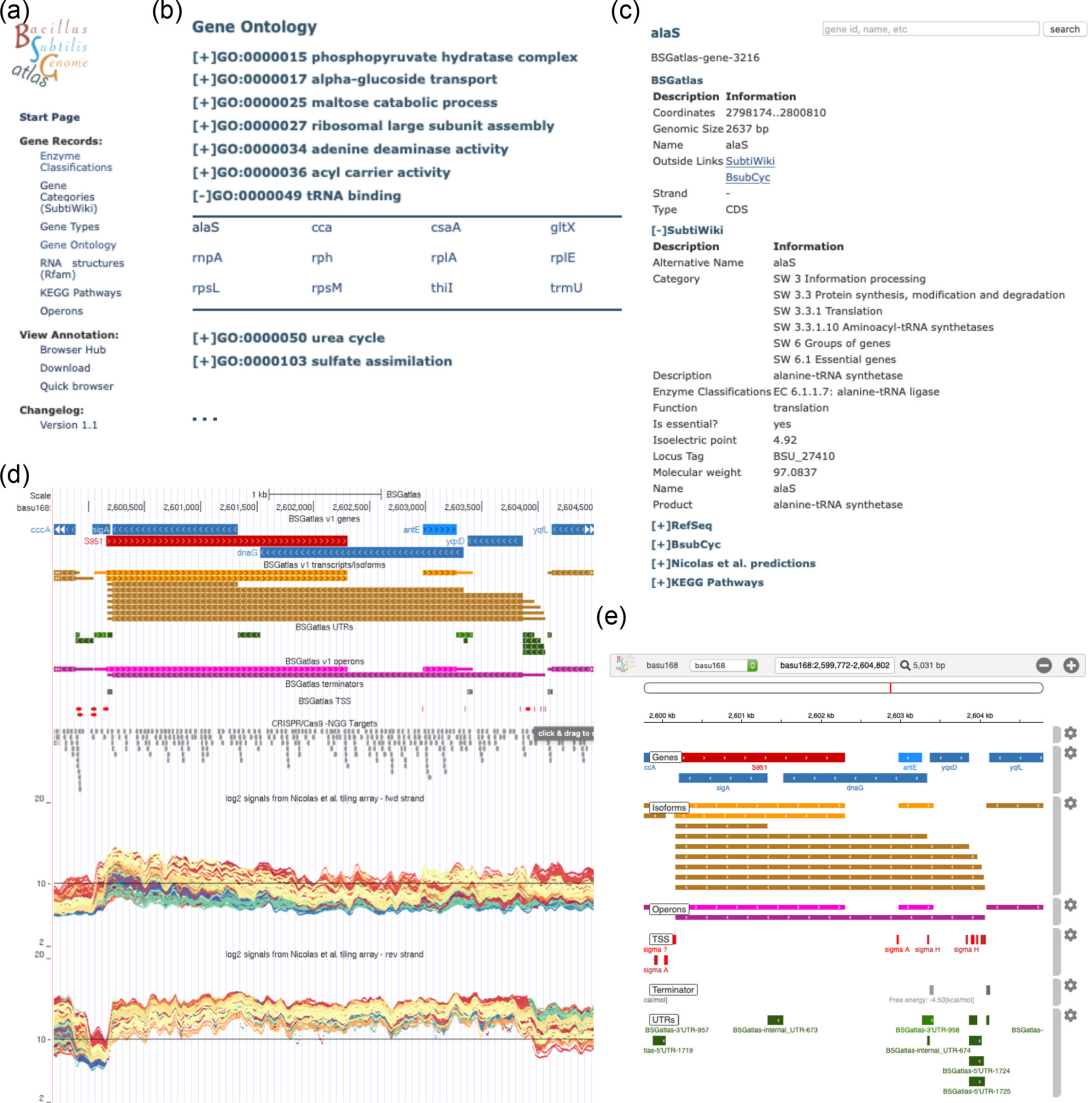
Illustration of the BSGatlas and its features. (a) The BSGatlas start page provides the main groups of navigational entries that lead to either the gene records, the search function or the various visualization options. (b) The gene record pages have the same structure across each classification system, these are gene types, enzyme classes or functional classification as listed in SubtiWiki or GO terms (a subset is shown in the figure). Each group is shown as a clickable entry, which list its associated genes. The gene names are shown as links to (c) detailed description pages. These show all meta-information we found for all genes and all other annotated entries, such as transcripts and operons. (d) UCSC genome browser. The user has the option to directly show the BSGatlas annotation as an assembly hub in the UCSC browser. Thus, they can also show their data, e.g*.* an RNA-seq experiment, right next to the annotation. Underneath the UCSC browser panel, a user can control details of what parts of the BSGatlas are shown. In addition, this includes the gene coordinates as they were originally annotated, which allows a closer investigation of the gene merging process. Here, a user can also opt-in to show the predicted CRISPR–Cas9 gRNAs and the signals from the tiling-array study. A click on the individual tracks shows more in-depth information on the guides and the tiling array colouration. (e) Quick browser. We provide a fast visualization directly on the BSGatlas main page, which allows a user to get a quick overview of the annotation without the need to leave the webpage. A click on any BSGatlas annotation redirects to the corresponding description page. (The browser is available at http://rth.dk/resources/bsgatlas/).

*Operon architecture.* Of the 2266 operons, 1396 were monocistronic and 865 polycistronic. We also considered the evolutionary context to related micro-organisms. It was of interest to compare operons we obtained to those of related micro-organisms; however, to our knowledge, only a few comprehensive genome-wide annotations are available based on high-resolution RNA-seq data. Therefore, we compared the operons to operon annotations in *Streptococcus pneumoniae* [77] and *E. coli* [10] (Fig. 2). BSGatlas annotates operons without alternative isoforms as simple, monocistronic operons in 45.5 % of all cases, and only 13.2 % as traditional, polycistronic. A total of 41.3 % are annotated as complex operons, meaning that they have alternative isoforms due to alternative TSSs and TTSs. In comparison, *E. coli* has 45 % simple, 19 % traditional and 36 % complex operons. *S. pneumoniae* has 47 % simple, 10 % traditional and 43 % complex operons. We computed the distribution of the number of genes, internal TSSs and internal TTSs per operon, and visually observed similar distributions between BSGatlas’ *B. subtilis* and *S. pneumoniae* (Fig. S compared to Warrier *et al.*’s figure 3b), although BSGatlas annotated more monocistronic operons without known TSSs or TTSs. Given that our inference is based on already curated TUs, we claim that our operon annotations are biologically reliable. We showed that the distribution of operon classes was comparable to other micro-organisms, which further supports that statement. A homology-based comparison of the annotations between the organisms would be needed for a definite statement about the phylogenetic conservation, but this would be outside of the scope of this annotation effort.

*Verification of UTR biotypes.* The 5′ and 3′ UTR annotations computed here resulted from matching TSS/TTS to the nearest downstream/upstream gene with a cut-off. We verified the approach and the resulting annotations by first confirming that the UTR annotations by Nicolas *et al.* [4] were still contained within (both in coordinates and type). Given that the number of our annotations was not only substantially larger but also solely based on a distance cut-off, we also checked for evidence of expression for the entire annotated region in the tiling-arrays signal (see next subsection). For the initial confirmation step, we identified pairs of annotations between the annotations computed here and those of Nicolas *et al.* that overlapped and checked for consistency in biotypes (Table S4). Overall, 95.6 % of all Nicolas *et al.* predicted 5′ UTRs (646 of 676) overlapped and agreed in biotype with our computed UTRs, although there were a few additional overlaps with other UTR types in the case of isoforms. The Nicolas *et al.* predicted 3′ UTRs are distinguished into UTRs with and without clear termination information. The agreement for 3′ UTRs with clear signals was 84.8 % (106 of 125). Non-surprisingly, only 46 % (57 of 124) of 3′ UTRs without clear termination information overlapped. These 57 3′ UTRs are predominately not annotated in the BSGatlas, because of the missing termination information. Nicolas *et al.* also annotated intergenic/intragenic regions of which 69.7 % (352 out of 505) had overlaps with our UTR annotations. Because these overlaps were relative to all UTR types (5′/3′/internal), we argue that the terminology intergenic/intragenic is unsuitable to fully annotate UTRs. Overall, 46 Nicolas *et al.* annotations overlap both a 5′ and a 3′ UTR in the BSGatlas (Table S4). We suspect two main reasons for this. On the one hand, the tilling-array study had a resolution limitation, which leads to difficulties in identifying separate UTR elements, such as described above. On the other hand, the TU annotation sources – that we used – were not available at the time-point of the Nicolas *et al.* milestone study. Yet, even with the availability of higher-resolution RNA-seq data, the lack of TU annotation hinders the prediction of internal UTRs and makes it a non-trivial endeavour to clearly distinguish them from two overlapping 5′/3′ UTRs of two separate operons/TUs [78]. The overlap comparison showed that 26.6 % (1421) of our 5335 obtained UTRs were not present in other resources and, thus, these might be new UTR annotations instead of refinements. In total, we annotated nearly four times more UTRs than the Nicolas *et al.* annotations.

*Transcription signal evidence.* In order to assess the reliability of the large number of novel UTR annotations and the BSGatlas in general, we checked to what extent these annotations had evidence of transcription. This was done by loading the transcription signals of the entire tiling-array signals of Nicolas *et al*. [4] Unfortunately, the normalization method used by Nicolas *et al.* is no longer available, yet it has been suggested that normalization for that specific array design might not be needed [66]. We computed the mean max log2 tiling-array signal coverages for each bp position across all conditions (ML2) of the BSGatlas annotations and those of Nicolas *et al*. For control purposes, we also included the ML2 for regions that were annotated neither in the BSGatlas nor in the work of Nicolas *et al.* (annotation gaps). The coding genes had the highest transcription signal where 90 % of coding annotations had ML2 >11.5 and 50 % had ML2 >14 (see ML2 distribution in Fig. S9). The 90 and 50 % ML2 percentiles for non-coding genes were 11.5 and 13.5, which are slightly lower than those of the coding genes. Furthermore, the level of overall transcription signals for the 5′ and internal UTR annotations proposed in the BSGatlas were about on the same coverage levels as the 5′ UTR annotations of Nicolas *et al.* and the non-coding genes according to the ML2 measure. Given the decrease of sequencing signal towards the 3′ end of transcripts, which are particular for all types of DNA-array chips [79], it is not surprising that the 3′ UTR annotations had an overall weaker signal strength: 90 % of the Nicolas *et al.* 3′ UTRs had ML2 >11.5 and 50 % had ML2 >12. The ML2 percentiles for the 3′ UTRs proposed in this study were 11 (90 %) and 13 (50 %). The ML2 coverages of Nicolas *et al.*’s intergenic/intragenic regions were between the ML2 values of 3′ and 5′ UTRs, which further emphasizes that these regions should be classified as separate types. However, 95 % of all annotations (from both BSGatlas and Nicolas *et al.)* have an ML2 far above 10.5. In comparison, 55 % of annotation gaps had an ML2 <10.5 and 85 % ML2 <11. Therefore, we suggest that the annotation proposed in this study has clear evidence of transcriptional activity. Surprisingly few annotation gaps (<5 %) had potential evidence of transcription (ML2 >12), which indicates potential for future annotation efforts; however, a more in-depth analysis of these regions and the individual tiling-array experiments is out of scope of this paper.

### Genome browser hub with enhanced information access

The UCSC genome browser provides the framework to easily visualize and share track information from sequencing experiments and annotation sources [80]. There is a *B. subtilis* genome browser in UCSC’s archaeal genome section [81], yet this hub has the same protein-centric focus as discussed earlier and a limited set of data tracks. Thus, we compiled our BSGatlas annotation as an assembly hub (Fig. 3d), thereby allowing users to investigate their data side by side with our annotation, without the need to install dedicated software on their machines. We set up the hub to allow searches for all genes by their names, synonyms and loci identifiers, including the alternatives and spelling variants available from all used resources. For each annotated gene, transcript, TSS, etc., we provide a detailed summary page that contains all meta-information that we retrieved from externally available resources (Fig. 3c). For each piece of meta-information, we indicate from which of the external resources it originated, and we link back to the external resources. Moreover, our hub contains the tracks for the resources used to create BSGatlas, meaning that users can compare the original annotation themselves. The genome browser also includes a table browser and a blat search option (which we enable through our server infrastructure) [82], which facilitates the easy download of data sets and identification of *B. subtilis* genomic sequences, respectively. To ease the navigation of the large number of annotation-tracks the BSGatlas provides, we grouped them. A user can interactively activate the display of groups in the genome browser, and they can select for each group individually their tracks of interest. On each detailed description page, we include a lightweight browser (based on the *igv.js* library [83]) (Fig. 3e), which allows a user to get a fast overview of the genomic context of an annotation. We also provide the annotation as a GFF3 download option (Fig. S1e) to facilitate offline visualization of the BSGatlas with programs such as IGV or IGB [83, 84]. We use a unified colour scheme across the different visualization options. The colour scheme indicates the gene types (protein, tRNA, rRNA, etc.) and the strand location. Additionally, we provide gene record information for the various gene types and gene sets; these are at the moment enzyme classifications, functional annotation with GO terms and SubtiWiki’s category system (Fig. 3a, b). Thus, the BSGatlas now offers access to gene records in a single combined resource, and users can compare the meta-information between the resources more easily. The BSGatlas can be accessed at: https://rth.dk/resources/bsgatlas/.

We included the tiling-array signals of the over a hundred conditions from the work by Nicolas *et al.* [4] for visualization in the genome browser. Given the large number of conditions and replicates (>250 tracks), we added a composite track that allows enabling/disabling individual experiments by users. The tracks are coloured according to the experiments, and replicates were kept separate. To improve readability, the browser shows the signals with a ‘smoothing window’ by default. A user can disable that smoothing in the browser.

### CRISPR gRNA design with polycistronic off-targets

Currently, no genome-wide gRNA libraries exist for *B. subtilis* that could tell the predicted efficiency or specificity of the different gRNA designs. When multiple target regions/gRNAs are possible for desired genome-edit purposes, computational assessment of alternative gRNAs could maximize the success rates prior to unnecessary experimental efforts. In the UCSC genome browser hub, we present all on-target regions in the *B. subtilis* genome that could be targeted with the CRISPR–Cas9 system. Each on-target (gRNA) is evaluated for its on-target efficiency and off-target potential (see Methods). In addition to these scores, for every gRNA, we list the potential off-target genomic regions for up to four mismatches (although up to six is used for the score computation). We also extended the list of putative gene off-targets by using the BSGatlas operon annotations of polycistronic operons, because the CRISPR complex has a demonstrated impact on the entire operon [48]. To our knowledge, no previous large scale gRNA predictions for the *B. subtilis* genome exist, in particular because of the lack of easy accessibility to operon annotations as are now possible with the BSGatlas. Overall, our CRISPRspec pipeline identified 378 050 guide sequences of 23 nt lengths (including PAM sequences). We only predict guides with NGG PAMs, but also consider off-targets with other PAMs (NGG, NAG and NGA). The specificity prediction indicates that 1792 gRNA have multiple on-target regions (exact sequence match). These potential multiple on-target positions are also listed in the browser. By default, the browser shows only guides with specificity CRISPRspec ≥7, a cut-off found to identify the specific guides in the CRISPRoff pipeline [47]. A user can choose to show all guides. All this information is highly beneficial to the community that is interested in CRISPR–Cas9 applications in *B. subtilis *, to minimize errors in genome-editing.

### Conclusion

*Bacillus* species are important for cell-based protein production and *B. subtilis* is probably the most studied Gram-positive bacteria. Although *B. subtilis* was sequenced for the first time more than 20 years ago [60], its full annotation has been lacking substantial information. In this study, we have made a significant step towards a far more complete annotation. We carefully merged the existing annotations from BsubCyc [35], SubtiWiki [4, 8], RefSeq [24], our Rfam scan [38, 39], and from a tiling-array and Term-seq study (Table 1). Besides the annotations for coding and non-coding genes and structured RNA, we integrated TSS, TTS and TU annotations. Taken together, these annotations implied novel UTR and transcript annotations. The combined effort resulted in a single atlas annotation for *B. subtilis* that comprises genes, transcripts and operons.

Despite that Nicolas *et al.* [4] annotated over 1500 non-coding annotations (both UTRs and ncRNA), the atlas led to an increase in the number of structured RNA elements and non-coding genes by 196, which are based on the Nicolas *et al.* tilling-array study (113), the Rfam screen [25] and riboswitches detected by the Term-seq experiment of Dar *et al.* [26]; 31 annotations were from a combination of these resources (see ‘Improvements in the number of ncRNA annotations’ sub-section). Moreover, we inferred a unified set of 3390 TSSs and 2566 TTSs, for which the external resources indicate a high curation level and resolution confidence in 43.8 % of all TTSs. The annotation of TSSs still lags substantially behind, as existing curations provide a high-resolution for only 20.8 % of TSSs. We combined information from TSSs and TTSs with known TUs to infer novel TUs and deduce full transcript annotations, which increased by nearly fivefold the number of 5′ and 3′ UTR annotations and doubled internal UTRs. These transcript annotations do not only provide transcriptional information for an unprecedented proportion of genes, they also give insight into the complex operon architecture of the genome. The gene annotations were generated by merging annotation from multiple annotation resources of different levels of confidence. Although our automized merging procedure should be sufficient for most users, we still show in the browser (optional tracks) the annotations as they were in the individual resources. These original annotations are important for being aware of specific use-cases in which a user needs to consider potential alternative annotations for a gene of interest. A comparison with the comprehensive study of transcription signals by Nicolas *et al.* further showed that our annotation, including the UTR annotations inferred by deduction from TSS/TTS information, are plausibly transcribed. However, such a comparison cannot substitute curated investigation, as those that have been summarized in DBTBS or BsubCyc.

In summary, we integrated the information currently available for *B. subtilis *, to our knowledge making the BSGatlas the most comprehensive genome and transcriptome annotation of its kind. In particular, our annotation contains more ncRNAs than existing resources and provides transcriptional annotation for nearly 93 % of all genes. Moreover, we have all the information easily accessible via the UCSC genome browser framework, allowing researchers to easily access, download and visualize the data. Thus, we anticipate that the BSGatlas will complement existing resources. The BSGatlas should also be useful in studying the function of the *Bacillus* genome; in particular for the investigation of non-coding genes and transcriptional relationships of genes in transcriptomic studies.

When generating the BSGatlas, we adhered to current standards on computational reproducibility. Thus, the methodology and their code implementation are re-usable not only for future versions of BSGatlas, but also for potential similar annotations of other bacteria. We also provide all computational results in public repositories for long-term availability. That availability is a requirement to allow it to be used in for instance RNA-seq studies, but it also allows programmatic access to the annotation. For instance, a researcher could easily set-up an own browser copy, or the information can be integrated into other databases.

## Supplementary Data

Supplementary material 1

Supplementary material 2
