# Supplementary material for: BSGatlas: a unified Bacillus subtilis genome and transcriptome annotation atlas with enhanced information access
Source: Microb Genom. 2021 Feb 4;7(2):000524. doi: 10.1099/mgen.0.000524 (PMC8208703; doi:10.1099/mgen.0.000524)
Supplement: Supplementary material 1 [file mgen-7-524-s001.pdf]

Supplementary Materials

BSGatlas: A unified *Bacillus subtilis* genome and transcriptome annotation atlas with enhanced information access

Adrian Sven Geissler[1], Christian Anthon[1], Ferhat Alkan[1,4], Enrique González-Tortuero[1a], Line Dahl Poulsen[2], Thomas Beuchert Kallehauge[3], Anne Breüner[3], Stefan Ernst Seemann[1], Jeppe Vinther[2], and Jan Gorodkin[1,*]

[1]Center for non-coding RNA in Technology and Health, Department of Veterinary and Animal Sciences, University of Copenhagen, Denmark

[2]Section for Computational and RNA Biology, Department of Biology, University of Copenhagen, Denmark

[3]Novozymes A/S, Bagsværd, Denmark

[4] Division of Oncogenomics, Netherlands Cancer Institute, The Netherlands

[*]gorodkin@rth.dk

[a] Current affiliation: School of Science, Engineering and Environment, University of Salford, United Kingdom

Table S1. Comparison of the individual gene annotation resources with the merged gene set. The topmost row contains the priority we assigned to that source, with a numerically lower value indicating higher confidence. Our general guideline behind the assigned confidence levels were to give higher priority (i) to resources annotating protein-coding genes, in order to avoid confusion of the overall clear boundaries of coding genes with the less clear boundaries of non-coding genes or structures, (ii) to the more recent resource, (iii) and to prefer expert curated or literature review resources over computational ones. We considered resources with equal priorities to be equally trustworthy, such that we joined their annotations with the union of the coordinates.

description	BSGatlas	RefSeq Coding	BsubCyc Coding	RefSeq Non-Coding	Rfam screen (conservative)	BsubCyc Non-Coding	Dar *et al* term-seq	Rfam screen (medium)	Nicolas *et al.* predictions
Resource Priority	–	0	1	2	2	3	3	4	4
Protein Coding Genes	4332	4325	4188	–	–	–	–	–	–
putative/predictions	79 (2%)	88 (2 %)	1210 (29 %)	–	–	–	–	–	–
Hypothetical status removed	–	9 (0 %)	1204 (29 %)	–	–	–	–	–	–
Merging refined coordinates of	–	1 (0 %)	49 (1 %)	–	–	–	–	–	–
Resource specific genes	–	144 (3 %)	8 (0 %)	–	–	–	–	–	–
Non-Coding RNAs	408	–	–	212	214	183	82	230	153
putative/predictions	137 (34 %)	–	–	22 (10 %)	0 (0 %)	28 (15 %)	0 (0 %)	0 (0 %)	153 (100 %)
known ncRNA types	–	–	–	190 (90 %)	214 (100 %)	155 (85 %)	82 (100 %)	230 (100 %)	0 (0 %)
ribosomal RNA (rRNA)	30 (7 %)	–	–	30 (14 %)	30 (14 %)	30 (16 %)	0 (0 %)	30 (13 %)	0 (0 %)
transfer RNA (tRNA)	86 (21 %)	–	–	86 (41 %)	86 (40 %)	86 (47 %)	0 (0 %)	86 (37 %)	0 (0 %)
small regulatory RNA (sRNA)	37 (9 %)	–	–	14 (7 %)	29 (14 %)	9 (5 %)	0 (0 %)	31 (13 %)	0 (0 %)
regulatory antisense RNA (asRNA)	8 (2 %)	–	–	3 (1 %)	2 (1 %)	2 (1 %)	0 (0 %)	4 (2 %)	0 (0 %)
riboswitch	104 (25 %)	–	–	55 (26 %)	63 (29 %)	26 (14 %)	82 (100 %)	73 (32 %)	0 (0 %)
self-splicing intron	3 (1 %)	–	–	0 (0 %)	1 (0 %)	0 (0 %)	0 (0 %)	3 (1 %)	0 (0 %)
other (ribozyme, SRP, tmRNA)	3 (1 %)	–	–	2 (1 %)	3 (1 %)	2 (1 %)	0 (0 %)	3 (1 %)	0 (0 %)
Coordinate refined	–	–	–	145 (68 %)	99 (46 %)	144 (79 %)	54 (66 %)	107 (47 %)	21 (14 %)
Hypothetical status removed	–	–	–	17 (8 %)	0 (0 %)	28 (15 %)	0 (10 %)	0 (0 %)	19 (12 %)
Reclassified as coding	–	–	–	–	–	–	–	–	1
Resource specific genes	–	–	–	10 (5 %)	–	–	27 (33 %)	8 (3 %)	133 (87 %)

Table S2. Comparison of the coordinates from each gene annotation resource (column 1) with the coordinates of the resulting genes after merging. Shown are the amount of refinements in bp (column 2) and the number of annotations that changed in interval bins (columns 3-7). The comparison under consideration of the gene lengths is shown in Figure S3.

Resource (Priority)	No difference	[1,10]	(10,50]	(50,100]	(100,250]	(250,500]
RefSeq Coding (0)	4,324	0	0	0	1	0
BsubCyc Coding (1)	4,139	18	13	12	6	0
RefSeq Non-Coding (2)	67	143	1	1	0	0
Rfam, conservative (2)	115	88	7	2	2	0
BsubCyc Non-Coding (3)	39	137	4	2	1	1
Dar *et al.* riboswitches (3)	28	6	15	27	6	0
Nicolas *et al.* predictions (4)	132	0	11	4	3	1
Rfam, medium (4)	123	92	10	2	2	1

Table S3. Distances in bp to the nearest annotation in the merged gene set compared to the Nicolas *et al.* predicted UTRs and intergenic regions. Overlapping closest genes are listed as such. Because Nicolas *et al.* separate UTRs into non-overlapping elements, we added the lengths of the fragments to better convey the length of the biological region.

distance to closest gene	3’ UTR	5’ UTR	intergenic	Internal UTR
Overlapping	24 (10%)	74 (11%)	18 (6%)	12 (6%)
[0, 100]	40 (16%)	210 (31%)	78 (24%)	81 (44%)
(100, 500]	87 (35%)	337 (50%)	166 (52%)	83 (45%)
(500, 1,000]	53 (21%)	40 (6%)	35 (11%)	8 (4%)
(1,000, 2,000]	38 (15%)	8 (1%)	18 (6%)	2 (1%)
2,000+	7 (3%)	7 (1%)	4 (1%)	0

Table S4. Overlap based comparison of our computed UTRs without those annotated by Nicolas *et al.* including their intergenic regions. Nicolas *et al*. annotated various types of UTRs (columns). The first row stated the number of annotations from Nicolas *et al*. The first column indicates the type of UTR annotations form the BSGatlas or a combination of UTRs (separated by comma) for which overlaps occurred (with single bp, no cut-off).

Overlapping BSGatlas UTRs	3'UTR	3'UTR (unclear termination)	5'UTR	intergenic	intragenic
# in Nicolas *et al.*	125	124	676	186	319
3'UTR	96	56	1	3	94
3'UTR,5'UTR	2	1	12	4	11
3'UTR,5'UTR,internal UTR	1	0	7	3	4
3'UTR,internal UTR	7	0	0	19	14
5'UTR	1	0	566	7	10
5'UTR,internal UTR	0	0	61	12	3
Internal UTR	0	1	2	104	64
without overlap	18	66	27	34	119

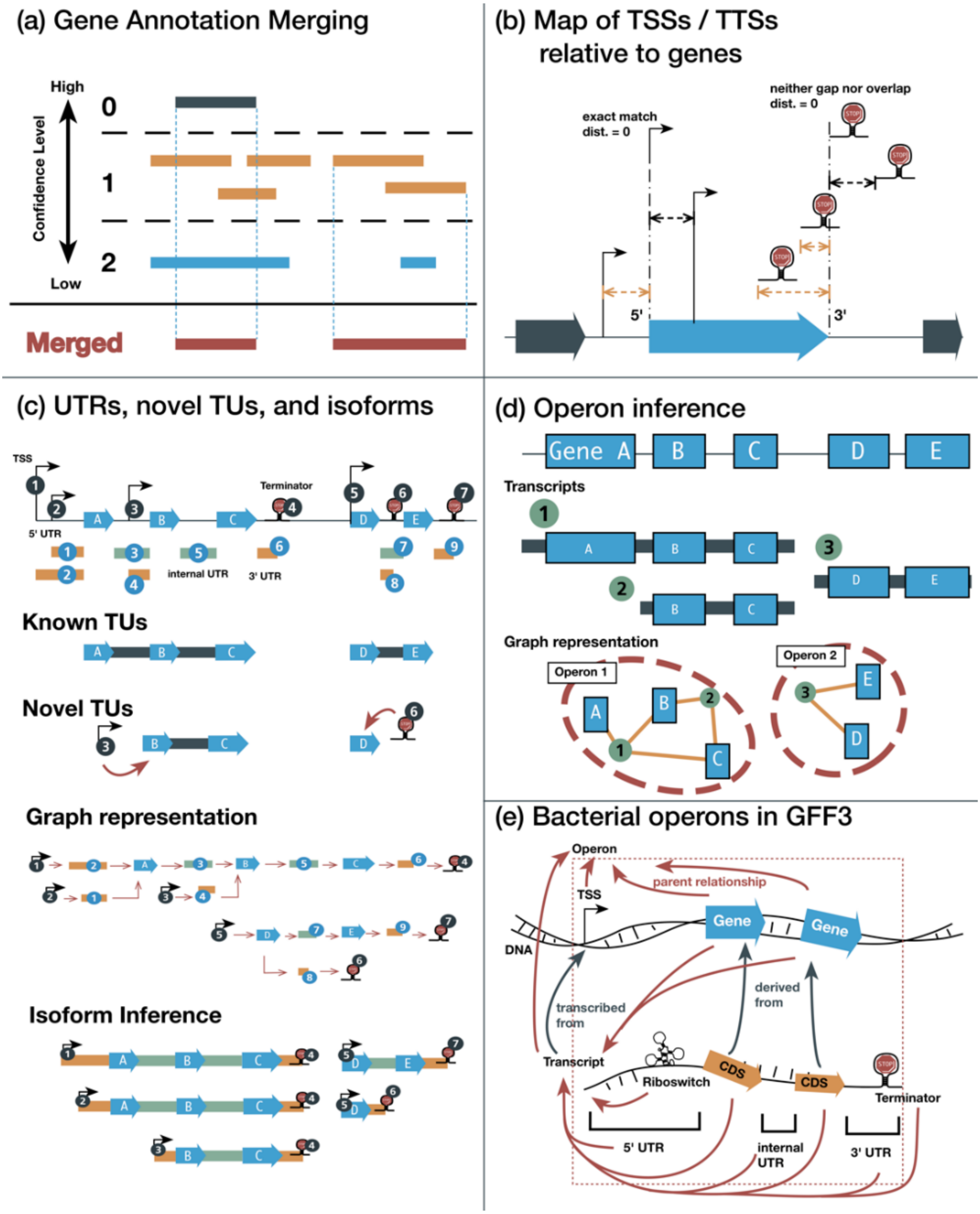

Figure S1. Outline of the annotation creation procedure. For the details refer to the main manuscript. (a) Gene annotation merging. Shown are two genes (red) for which the annotation resources provide differing coordinates (blue, orange, black). The merged coordinates are taken from the resource with the highest priority (left), or the union if there are multiple (right). (b) Distances that are used to determine the transcription start sites (TSSs) and terminator sites (TTSs) map. The TSS (arrow) distances are relative to the 5' end of a gene, for a TTS (stop sign) to the 3'. Instead of a single nucleotide position, TTSs annotated a region that forms the terminating hairpin, such that the distances are computed as shown. The orange highlighted distances are notated as a negative value. (c) Computation

of untranslated regions (UTRs), novel transcriptional units (TUs), and transcripts. Given a TSS/TTS map (arrow, stop sign, black numbers), 5' and 3' UTRs (orange with blue numbers) were placed in the space between them and the associated up-/down-stream gene (blue arrow). Internal UTRs (green with blue numbers) were implied by known TUs (black bar with gene regions highlighted). Novel TUs are implied by a TSS or TTS that is associated with a gene, which is either not the first or last gene in direction of transcription. Each TSS, TTS, UTR, gene is a unique element (colored numbers) that is present as a node in the directed transcription path. The full isoform list is inferred from all paths between TSSs and TTSs, which we derived from a graph. (d) Operon inference. We derived operons by finding connected components (red circles) in a graph with the transcripts (green numbers) and genes (blue) as nodes and edges (orange) indicating which genes are transcribed by which transcript. (e) Bacterial operons in GFF3. The GFF3 format models bacterial operons as shown: Each operon/UTR/gene/structure is an entry in the file, although each gene also has an extra entry to represent the transcribed region. The relationships between the entries are noted as indicated by the arrows.

```
Input:

• n ∈ ℤ⁺ gene annotations N = {x₁, …, xₙ}
• Each annotation has a start/end position, a strand, and a
  putative biotype
• Jaccard Index of two annotations i, j ∈ ℤ⁺ is JI(xᵢ, xⱼ)
  Note: For same strand overlapping annotations JI > 0
• Priority of the resource an annotation comes from p(xᵢ)

Merging procedure:
Let E be an empty set
// Investigate all pair-wise overlaps
For all i, j ∈ ℤ⁺ with i < j and JI(xᵢ, xⱼ) > 0:
     If xᵢ and xⱼ are a riboswitch and a coding sequence:
          // do not consider for merging
          continue
     If JI(xᵢ, xⱼ) ≥ 0.8:
          Add (xᵢ, xⱼ) to E
     If both xᵢ and xⱼ are non-coding annotations:
          If JI(xᵢ, xⱼ) ≥ 0.5 :
               Add (xᵢ, xⱼ) to E
          If annotation xᵢ fully contains xⱼ or vice versa:
               Add (xᵢ, xⱼ) to E
Let G(N, E) be an undirected graph
Let R be an empty set
For all connected components Cₖ of G:
     Let Cₖ := {x'₁, …, x'ₘ} be the annotations in the component
     //Compute max priority and the corresponding genes
     Let pmax := max({p(x'ᵢ) : x'ᵢ ∈ Cₖ})
     Let cmax := {x'ᵢ : x'ᵢ ∈ Cₖ if p(x'ᵢ) = pmax}
     // The merged annotation is the union of all annotations
     // of same priority
     Let r be annotation with
     * start(r) := min({start(x'ᵢ) : x'ᵢ ∈ cmax})
     * end(r) := max({end(x'ᵢ) : x'ᵢ ∈ cmax})
     * strand(r) := {strand(x'ᵢ) : x'ᵢ ∈ cmax} // is single value
     Add r to R
Return R
```

Figure S2: Pseudo-code describing in detail how the gene annotations were merged.

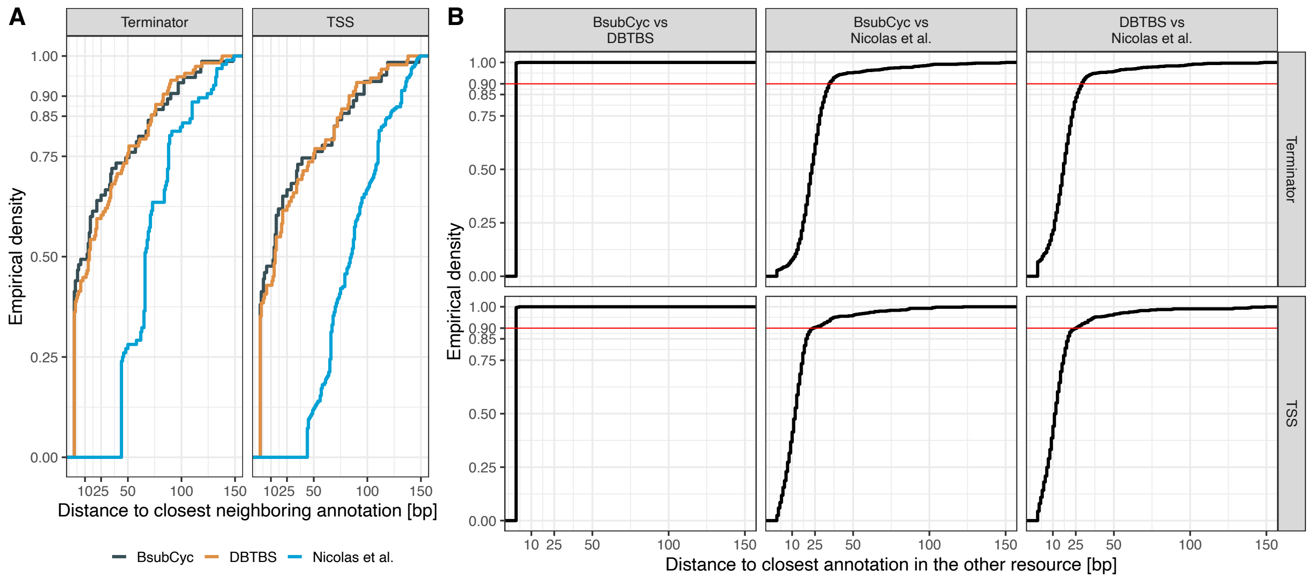

Figure S3. Shown are empirical cumulative distribution of distances for (A) two closest neighboring pairs of two TSS (left) or terminators (right) annotations within the resources BsubCyc (black), DBTBS (orange), and Nicolas *et al.* (light blue). (B) Distribution of closest pair of annotation between two resources (columns) for terminator and TSS annotations (rows). The red horizontal line indicates the 90% of annotations threshold.

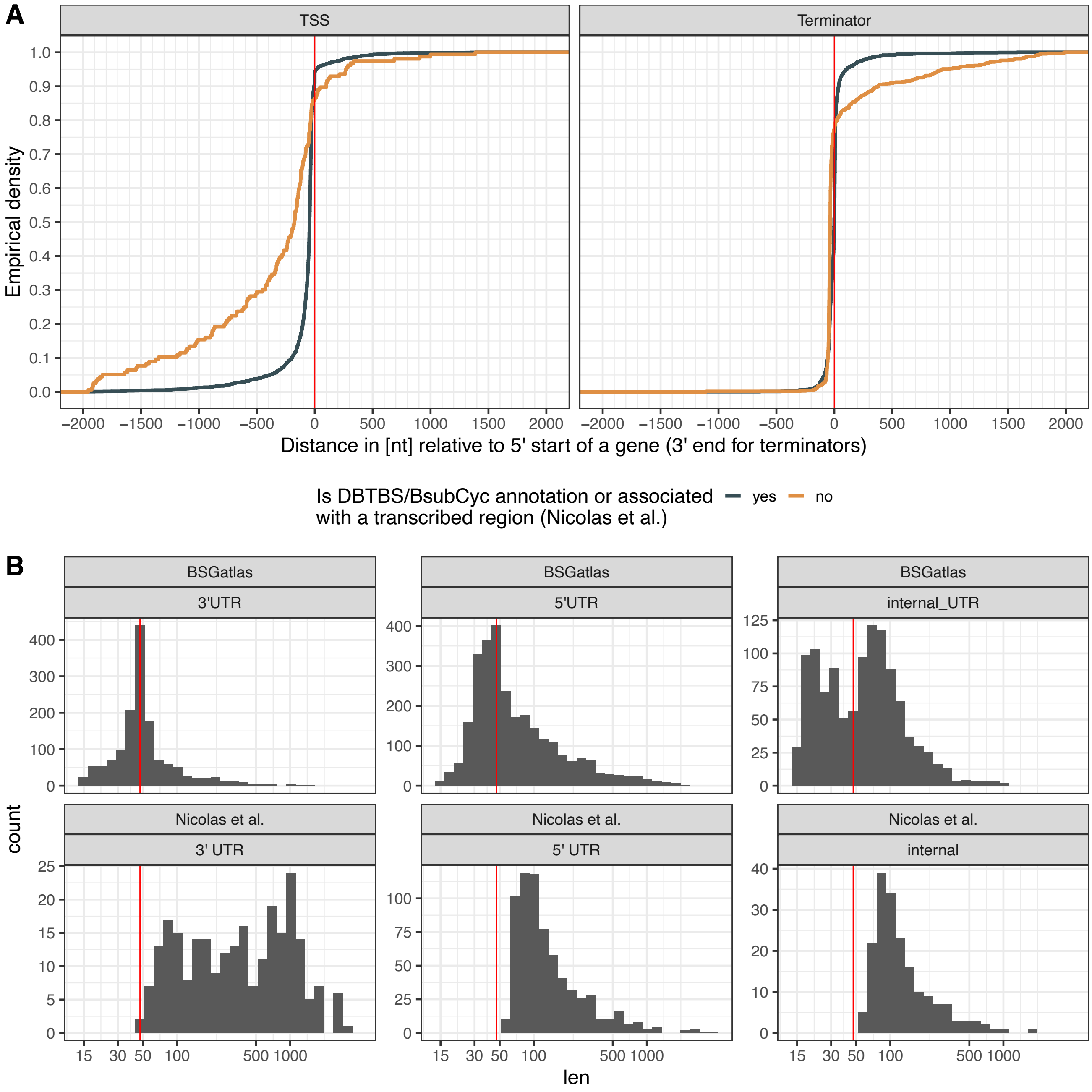

Figure S4. (A) Cumulative distribution of TSSs and TTSs relative to the closest 5'/3' end of genes. The red vertical lines represent the genes 5'/3' position, with negative distances indicating a before/upstream TSS/Terminator position. The distribution is separated by TSS/terminator annotations that are from DBTBS/BsubCyc and Nicolas et al. with an associated transcribed regions (blue) or without. (orange). (B) Distribution of lengths of our obtained UTRs in comparison to those found in Nicolas *et al.*'s tiling-array study. The UTRs of the latter resources have a minimal length of 47, which is indicated with the red line.

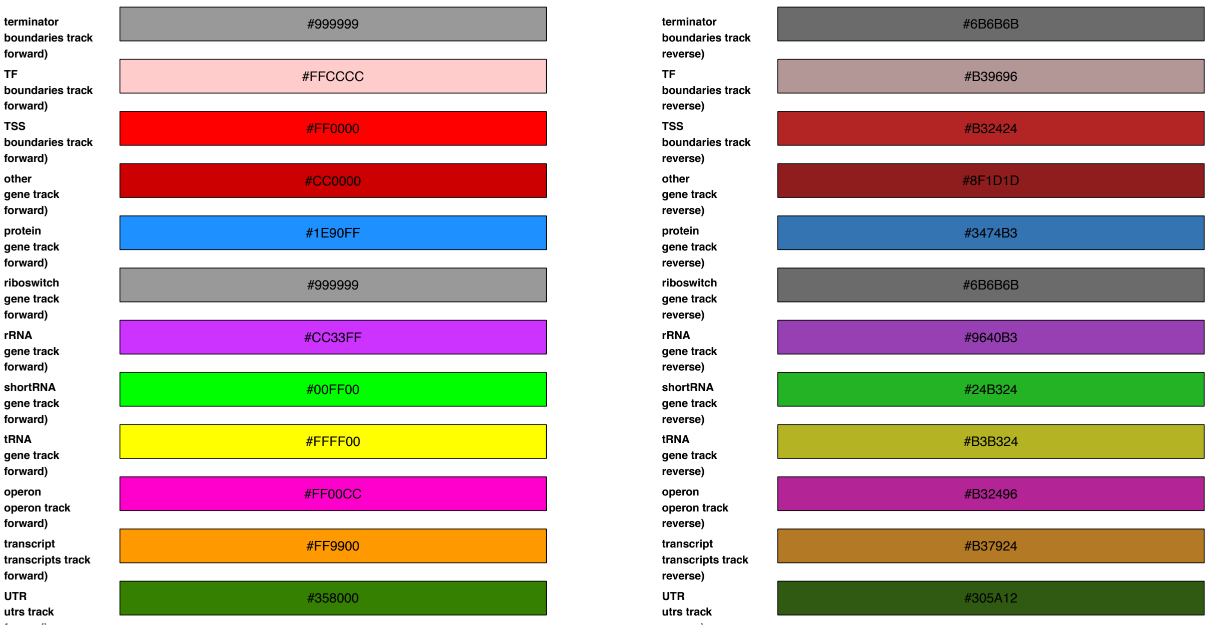

Figure S5. The color scheme for each type of the different annotated bio types (genes, structures, binding sites). Elements that are on located on the reverse strand are shown in a slightly darker color. We use this color coding across the different annotation visualizations that we offer in the UCSC browser hub, the GFF3 file, and the quick browser on the gene detail pages. Similar looking pairs of color or possibly for color blindness disadvantageous were avoided by putting these on separate browser tracks.

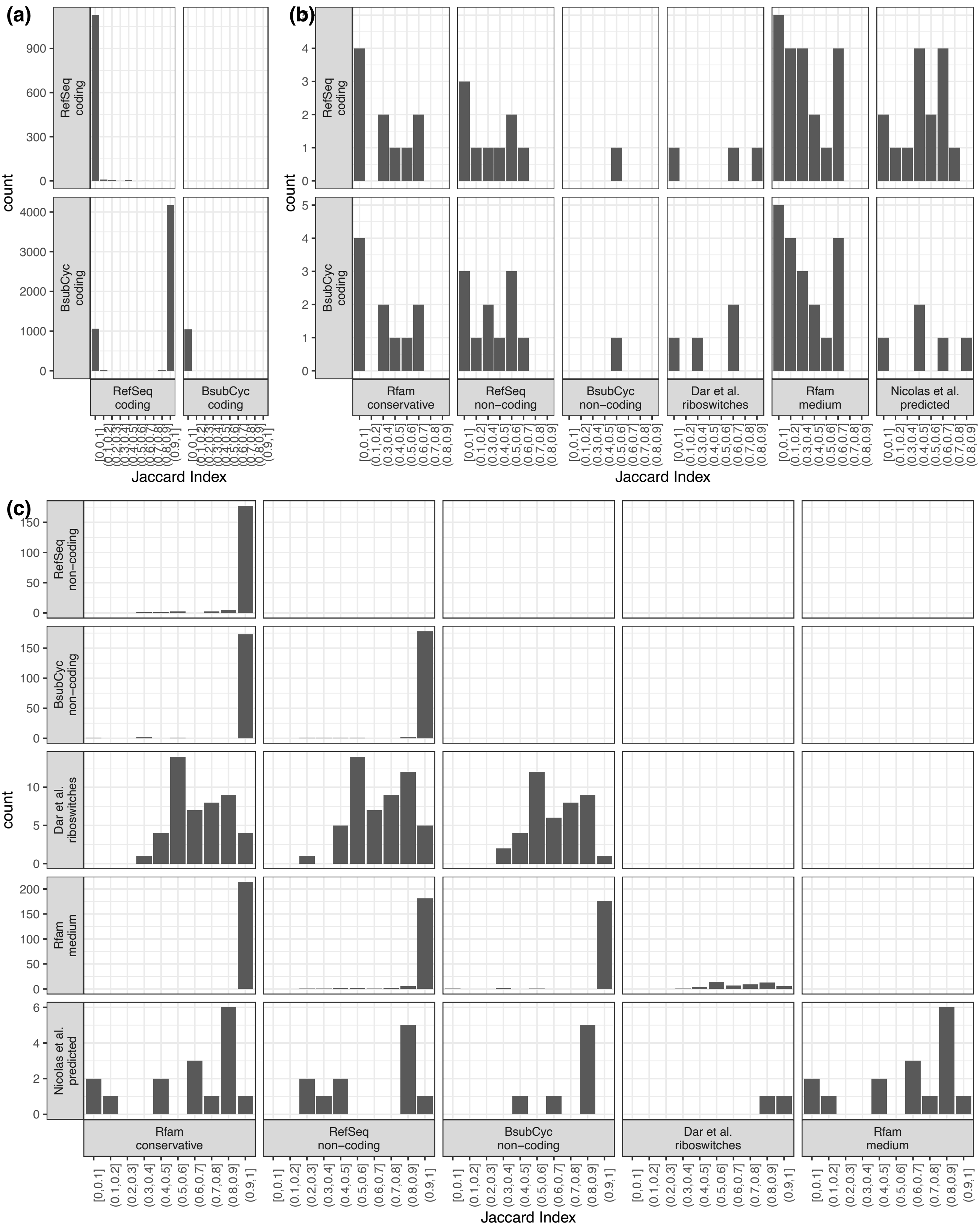

Figure S6. Distribution of Jaccard Indices between all overlapping pairs of genes from the collective annotation, separated by resource and (a) coding-coding gene pairs, (b) coding and non-coding, (c) non-coding – non-coding gene pairs.

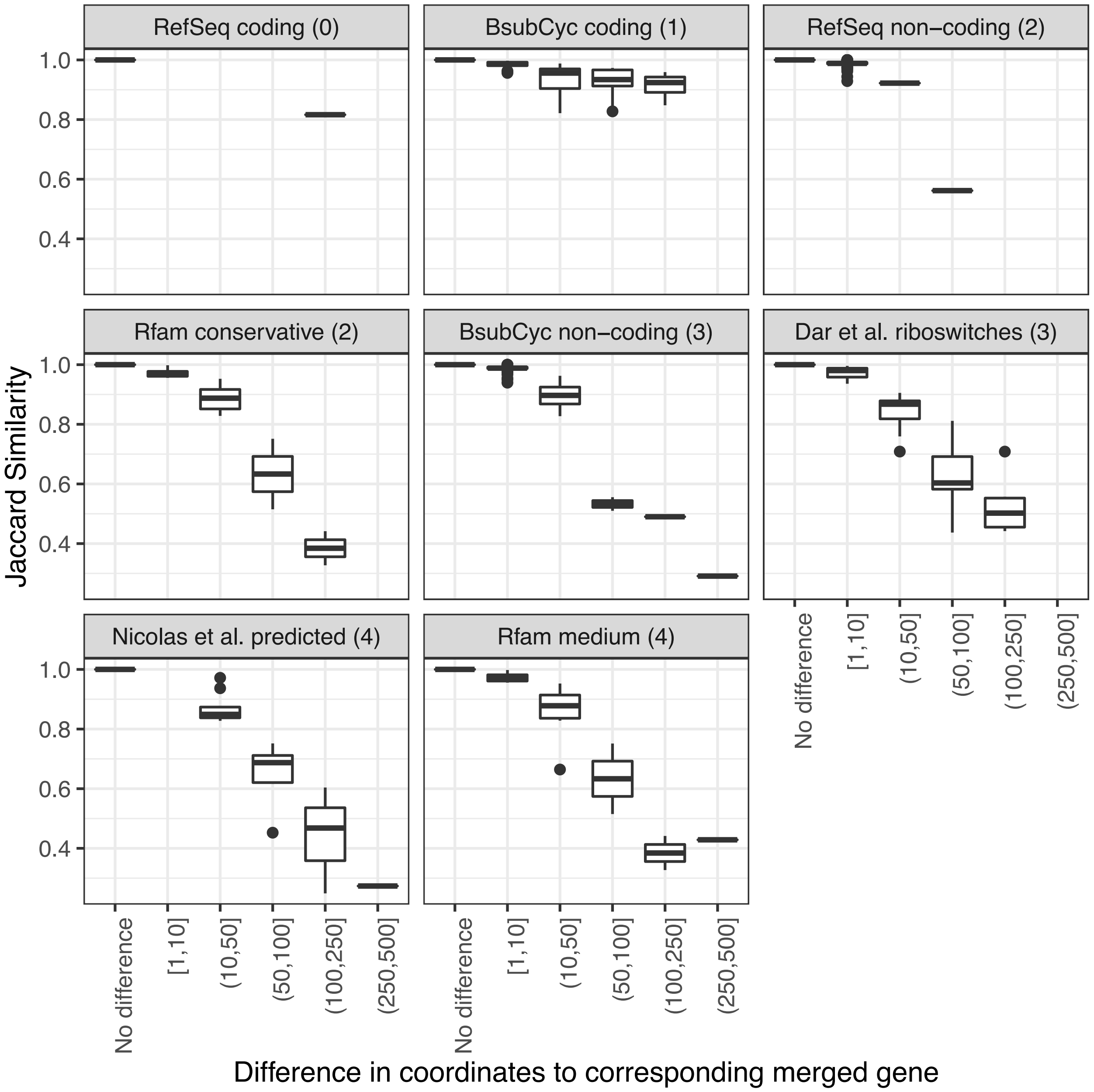

Figure S7. Comparison of the coordinates from each gene annotation resource with those from resulting genes after merging. Shown are the distributions of Jaccard similarity for various ranges of absolute coordinate differences in nucleotides. The numbers of how often a refinement in absolute numbers occurred are stated in Table S2.

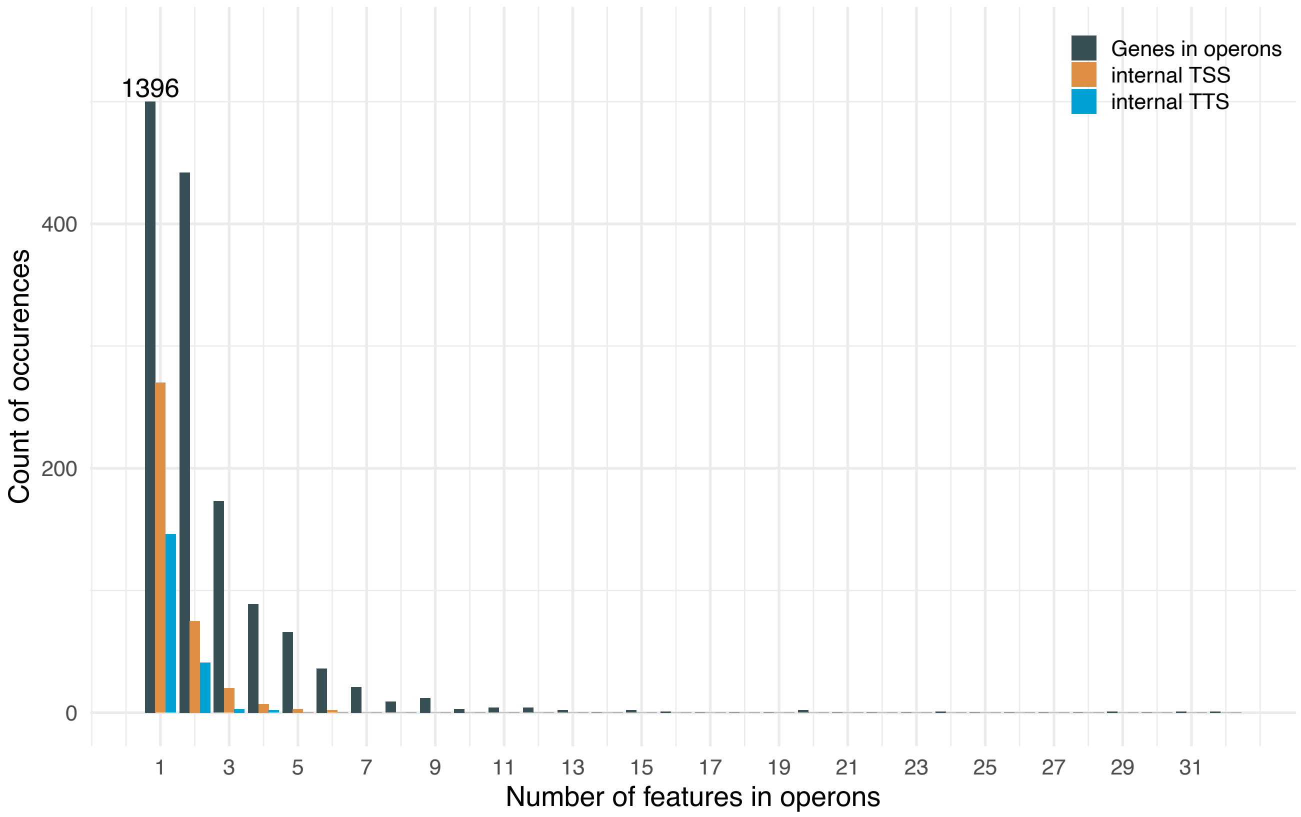

Figure S8. Distributions of the various features, such as the number of genes and internal TSSs / TTSs, for our computed operons in *B. subtilis*.

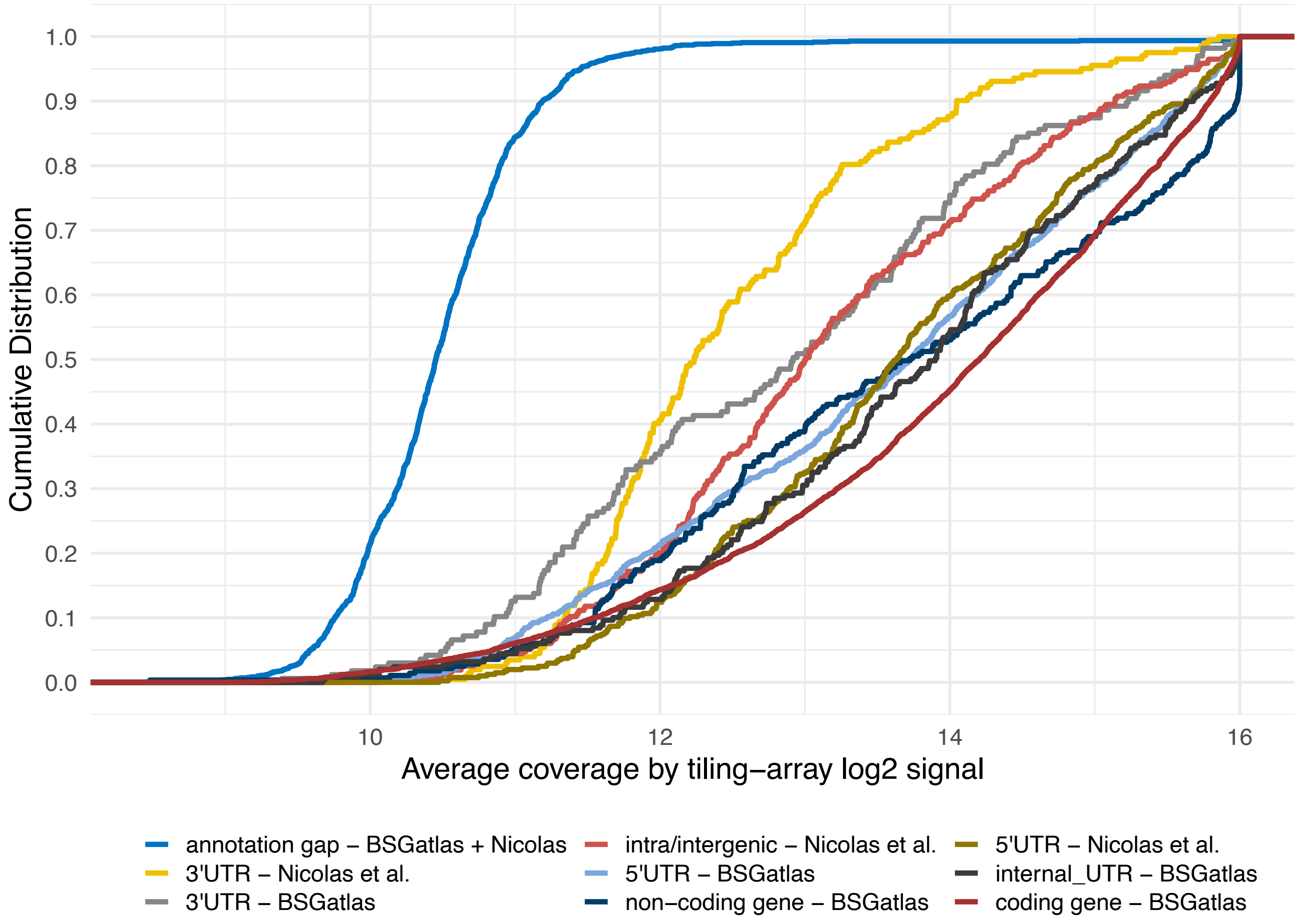

Figure S9. Coverage of annotations by tiling-array signal. We computed for various annotations (colors in legend) the average coverage by the maximal log2 of the Nicolas *et al.* tiling-array (see methods). Shown are the cumulative distribution of these average coverages. For control purposes we also added the average coverage of gaps in the BSGatlas (regions without annotation).
